# Structure of the Complete Dimeric Human GDAP1 Core Domain Provides Insights into Ligand Binding and Clustering of Disease Mutations

**DOI:** 10.3389/fmolb.2020.631232

**Published:** 2021-01-27

**Authors:** Giang Thi Tuyet Nguyen, Aleksi Sutinen, Arne Raasakka, Gopinath Muruganandam, Remy Loris, Petri Kursula

**Affiliations:** ^1^ Faculty of Biochemistry and Molecular Medicine and Biocenter Oulu, University of Oulu, Oulu, Finland; ^2^ Department of Biomedicine, University of Bergen, Bergen, Norway; ^3^ VIB-VUB Center for Structural Biology, Vlaams Instituut voor Biotechnologie, Brussels, Belgium; ^4^ Department of Bioengineering Sciences, Structural Biology Brussels, Vrije Universiteit Brussel, Brussels, Belgium

**Keywords:** protein structure, ganglioside-induced differentiation-associated protein 1, Charcot-Marie-Tooth disease, oligomeric state, fatty acid, membrane protein

## Abstract

Charcot-Marie-Tooth disease (CMT) is one of the most common inherited neurological disorders. Despite the common involvement of ganglioside-induced differentiation-associated protein 1 (GDAP1) in CMT, the protein structure and function, as well as the pathogenic mechanisms, remain unclear. We determined the crystal structure of the complete human GDAP1 core domain, which shows a novel mode of dimerization within the glutathione S-transferase (GST) family. The long GDAP1-specific insertion forms an extended helix and a flexible loop. GDAP1 is catalytically inactive toward classical GST substrates. Through metabolite screening, we identified a ligand for GDAP1, the fatty acid hexadecanedioic acid, which is relevant for mitochondrial membrane permeability and Ca^2+^ homeostasis. The fatty acid binds to a pocket next to a CMT-linked residue cluster, increases protein stability, and induces changes in protein conformation and oligomerization. The closest homologue of GDAP1, GDAP1L1, is monomeric in its full-length form. Our results highlight the uniqueness of GDAP1 within the GST family and point toward allosteric mechanisms in regulating GDAP1 oligomeric state and function.

## Introduction

Mutations in the *GDAP1* gene, coding for the ganglioside-induced differentiation-associated protein 1 (GDAP1), are associated with several forms of Charcot-Marie-Tooth disease (CMT), which is one of the most common inherited neurological disorders, affecting one in 2,500 people (Baxter et al., 2002; Cuesta et al., 2002; Auranen et al., 2013). GDAP1, a 358-amino-acid mitochondrial outer membrane (MOM) protein regulating the mitochondrial network, is highly expressed in neurons and less in Schwann cells (Niemann et al., 2005; Pedrola et al., 2005). GDAP1 contains two domains similar to the N- and C-terminal domains of glutathione (GSH) S-transferases (GST) (GST-N and GST-C, respectively), a hydrophobic domain (HD), and a transmembrane domain (TMD) (Huber et al., 2016). GDAP1 shares only ∼20% sequence identity with canonical GSTs. Several GDAP1 constructs were previously assayed against a group of GST substrates, but no GSH-dependent activity or binding to GSH was detected (Shield et al., 2006; Googins et al., 2020). However, a previous study suggested that GDAP1 has theta-class-like GST activity *in vitro*, which is regulated by the HD in an autoinhibitory manner (Huber et al., 2016).

Purified GDAP1 overexpressed in bacteria and insect cells forms dimers in solution, as shown by glutaraldehyde crosslinking and size-exclusion chromatography (SEC) (Shield et al., 2006; Huber et al., 2016). Endogenous GDAP1 of human neuroblastoma SHSY5Y cells was detected in both dimeric and monomeric forms (Pedrola et al., 2005). The GDAP1 dimer disappeared under reducing conditions, implying that dimerization would be mediated *via* disulfide bonds. Contrary to these observations, the first crystal structure of the GDAP1 core domain, from a truncated construct lacking the large GDAP1-specific insertion, suggested that GDAP1 is monomeric (Googins et al., 2020). In light of these data, the GDAP1 insertion could play a role in GDAP1 dimerization and function.

GDAP1 functions in regulating the mitochondrial network by inducing fragmentation of mitochondria without inducing apoptosis. This fission activity is significantly reduced for CMT-related GDAP1 mutations (Niemann et al., 2005). Recessive mutations in GDAP1 are associated with decreased fission activity, whereas dominant mutations induce impairment of mitochondrial fusion, increased production of reactive oxygen species (ROS), and susceptibility to apoptotic stimuli (Niemann et al., 2009). To regulate various cellular processes, mitochondria use Ca^2+^ uptake and release to modulate cytosolic Ca^2+^ signaling (De Stefani et al., 2016). GDAP1 deficiency reduces Ca^2+^ inflow through store-operated Ca^2+^ entry (SOCE) activity and endoplasmic reticulum (ER) Ca^2+^ levels (Barneo-Muñoz et al., 2015). In the presence of Ca^2+^ or Sr^2+^, long-chain saturated α,ω-dioic acids, including hexadecanedioic acid (HA), can induce cyclosporin A-insensitive permeability of the inner membrane of liver mitochondria (Dubinin et al., 2013).

The paralogous GDAP1-like protein 1 (GDAP1L1) shares a 55% sequence identity with GDAP1, and the HD and TMD are conserved (Marco et al., 2004). The HD and TMD are the targeting domains of GDAP1 for function in mitochondrial fission (Wagner et al., 2009); however, GDAP1L1 is mainly cytosolic (Niemann et al., 2014). GDAP1L1 can induce mitochondrial fission in the absence of GDAP1, implying that it may compensate for GDAP1 loss in the central nervous system (Niemann et al., 2014).

Here, we describe the crystal structure of the complete dimeric GDAP1 core domain, and based on its unique mode of dimerization, we propose a model for full-length GDAP1 on the MOM. We also provide a low-resolution model for monomeric GDAP1L1 based on small-angle X-ray scattering (SAXS) data. As no GST activity was detected for GDAP1, a metabolite library was screened for GDAP1 binding partners. We find that HA binds to GDAP1 and affects its stability, conformation, and oligomerization. The HA binding site in the crystal structure of GDAP1 is located close to the CMT-linked residue cluster and the membrane-binding surface.

## Materials and Methods

### Chemicals

Chemicals were from Sigma-Aldrich unless otherwise stated. Crystallization screens were from Molecular Dimensions. The Human Endogenous Metabolite Compound Library was from MedChemExpress.

### Cloning, Expression, and Purification

The open reading frame (ORF) of full-length human GDAP1 isoform 1 (UniProt ID: Q8TB36) was ordered from DNA2.0 as a synthetic codon-optimized gene for bacterial cytosolic expression in the pJ201 vector. The C-terminally truncated GDAP1∆295-358, GDAP1∆319-358, and GDAP1∆303-358 constructs were generated by PCR and transferred into the pDONR221 entry vector using Gateway^®^ technology-based site-specific recombination (Invitrogen). An N-terminal Tobacco Etch Virus (TEV) protease digestion site was included in each construct.

For structural and biochemical characterization, GDAP1 constructs were transferred into pTH27 (Hammarström et al., 2006) and pDEST-Trx (Tsunoda et al., 2005) vectors, which encode for N-terminal His_6_ and thioredoxin tags, respectively. Point mutations were generated using site-directed mutagenesis PCR (Shenoy and Visweswariah, 2003). The ORF of full-length GDAP1L1 was purchased in the pET28a (+)-TEV vector, containing a TEV protease cleavage site and a His_6_ tag (Genscript). All constructs were verified with DNA sequencing of both strands.

Recombinant protein expression was done using *E. coli* BL21 (DE3) in ZYM-5052 auto-induction medium (Studier, 2005). Selenomethionine-substituted (SeMet) protein was expressed using *E. coli* B834 (DE3) in SelenoMet™ -media (Molecular Dimensions) (Ramakrishnan et al., 1993).

The soluble recombinant protein was captured on a Ni^2+^-NTA affinity resin by gravity flow (Thermo Fisher Scientific). Unbound proteins were washed with 25 mM HEPES, 300 mM NaCl, 2% (v/v) glycerol, and 25 mM imidazole (pH 7.5). The protein was eluted with an identical buffer, with imidazole at 250 mM. The affinity tag was cleaved with a 1:20 molar ratio of TEV protease (16 h, +4 °C). The His_6_ tag and TEV protease were then removed by another Ni^2+^-NTA affinity step. SEC was performed on a Superdex 200 or Superdex 75 10/300 GL increase column (GE Healthcare) using 25 mM HEPES (pH 7.5), 300 mM NaCl (SEC buffer) as eluent. An anion exchange chromatography (IEX) step was added for GDAP1L1, using a HiTrap HP Q XL column (GE Healthcare). GDAP1L1 was eluted using a linearly increasing gradient up to 1 M NaCl in 30 mM Tris (pH 7.9). Peak fractions were analyzed with SDS-PAGE, and Coomassie-stained bands were analyzed using a Bruker UltrafleXtreme matrix-assisted laser desorption/time-of-flight mass spectrometer (MALDI TOF-MS). Tryptic peptides extracted from the gel were identified by searching NCBI and SwissProt databases using BioTools2.2 (Bruker).

### Crystallization, Data Collection, and Structure Determination

Crystallization was done using vapor diffusion at +4 °C. Protein and mother liquor drops were applied using a Mosquito LCP (TTP Labtech) nano-dispenser. The protein concentration was between 5–25 mg/ml in SEC buffer. Apo GDAP1∆303-358 was crystallized in 0.2 M magnesium formate, 20% (w/v) PEG 3350. GDAP1∆303-358 crystals with HA were obtained by co-crystallization with 1 mM HA (2% (v/v) EtOH as solvent), using 0.1 M succinic acid, 15% (w/v) PEG3350 as mother liquor. SeMet-GDAP1 crystals were grown in 0.2 M ammonium formate, 20% (w/v) PEG3350. Crystals were briefly soaked in 30% (v/v) glycerol before flash-freezing in liquid N_2_. Data collection was conducted on the synchrotron beamlines P11 (DESY, Hamburg, Germany), I24, and I04 (Diamond Light Source, Didcot, United Kingdom), at 100 K (Table 1).

**TABLE 1:** Diffraction data processing and refinement statistics.

Protein	SeMet GDAP1	Apo GDAP1	GDAP1-HA complex
Data collection			
Beamline	I04/Diamond	P11/PETRA III	I24/Diamond
Detector	Eiger2 XE 16M	Pilatus 6M	Eiger2 XE 16M
X-ray wavelength (Å)	0.9789	1.0332	0.9795
Space group	P2_1_2_1_2_1_	P2_1_2_1_2_1_	P2_1_2_1_2_1_
Unit cell dimensions a, b, c (Å)	72.9, 115.9, 116.2	72.8, 115.9, 116.6	73.0, 114.9, 116.9
α, β, γ (°)	90	90	90
Resolution range (Å)	30–3.17 (3.39–3.17)	50–2.80 (2.90–2.80)	50–2.20 (2.279–2.20)
No. unique reflections	16,992 (2,964)	24,884 (2,457)	50,525 (4,965)
Completeness (%)	99.0 (97.4)	99.6 (99.7)	99.7 (99.6)
Anom. Completeness (%)	99.1 (96.5)		
Redundancy	13.4 (13.0)	2.0 (2.0)	6.6 (6.6)
Anom. Redundancy	7.2 (6.9)		
CC_1/2_ Anom	0.633 (0.036)		
R_sym_ (%)	13.0 (70.3)	4.0 (36.2)	7.4 (128.6)
R_meas_ (%)	14.0 (75.9)	5.7 (51.19)	8.1 (139.6)
<I/σI>	15.1 (3.6)	11.55 (2.04)	12.72 (1.54)
CC_1/2_ (%)	99.9 (94.0)	99.8 (87.1)	99.8 (65.7)
Wilson *B* (Å^2^)	67.7	57.0	53.9
Structure refinement			
R_cryst_/R_free_ (%)		24.3/26.2	20.2/23.0
RMSD bond lengths (Å)		0.002	0.015
RMSD bond angles (°)		0.46	1.96
Molprobity score		0.88	1.84
Ramachandran favored/outliers (%)		97.6/0.2	97.8/0.4
PDB ID		7ALM	7AIA

Data were processed and scaled with XDS (Kabsch, 2010) and AIMLESS (Evans and Murshudov, 2013). Phases were obtained from SeMet data (Sutinen et al., 2020) with single-wavelength anomalous dispersion (SAD) using the Crank2 pipeline (Skubák and Pannu, 2013), and the initial model building was done using BUCCANEER (Cowtan, 2006). Molecular replacement, refinement, and structure validation were done using Phenix (Adams et al., 2010; Liebschner et al., 2019) and CCP4 (Winn et al., 2011). The models were refined using phenix.refine (Afonine et al., 2012) or Refmac5 (Murshudov et al., 2011) and rebuilt using COOT (Emsley et al., 2010). The GDAP1-HA complex structure was solved through molecular replacement with Phaser (McCoy et al., 2007), using GDAP1 as a model. The structures were validated using MolProbity (Chen et al., 2010) and deposited at the PDB with entry codes 7ALM (apo) and 7AIA (HA complex). Diffraction images for the SeMet dataset (Sutinen et al., 2020) used to solve the structure were deposited at Zenodo (http://doi.org/10.5281/zenodo.3988189).

### Bioinformatics and Modeling

Structure visualization was done with PyMOL (http://www.pymol.org) and Chimera (Pettersen et al., 2004). Schematic views of the interactions were generated with LIGPLOT (Wallace et al., 1995). Electrostatic surfaces were calculated with APBS and PDB2PQR (Unni et al., 2011). Accessible surface area and intermolecular interaction calculations were done using AREAIMOL (Winn et al., 2011) and PISA (Krissinel, 2015). Structural homology searches were done using PSI-search (Li et al., 2012) and SALAMI (Margraf et al., 2009), and selected sequences were aligned with T-COFFEE (Notredame, 2010; Di Tommaso et al., 2011). Manual editing of the sequences was done using Genedoc and ESPRIPT3.0 (Nicholas, 1997; Gouet et al., 1999). Full-length GDAP1 modeling within a membrane was done using COOT and YASARA (Krieger and Vriend, 2015).

### Small-Angle X-Ray Scattering

The GDAP1 monomer and dimer species were separated and analyzed with SEC-SAXS. Prior to the experiment, the samples were dialyzed against 25 mM HEPES pH 7.5, 300 mM NaCl and centrifuged at >20,000 *g* for 10 min at +4 °C to remove aggregates. SAXS experiments were performed on the P12 beamline (Blanchet et al., 2015) (EMBL/DESY, Hamburg, Germany), the SWING beamline (David and Pérez, 2009) (SOLEIL synchrotron, Saint Aubin, France), and the B21 beamline (Cowieson et al., 2020) (Diamond Light Source, Didcot, United Kingdom).

The data were collected over an s-range of 0.003–0.5 Å^−1^ (s* = 4π* sin*(θ)/λ*, where 2θ is the scattering angle) at a fixed temperature (+15 °C). 50 µL of a protein sample at 8.5–10 mg/ml were injected into a BioSEC3-300 (Agilent) or Superdex 75 10/300 GL increase column (GE Healthcare) and eluted at a flow rate of 0.2 ml/ml or 0.5 mg/ml. Data reduction to absolute units, frame averaging, and subtraction were performed using Foxtrot (David and Pérez, 2009) or CHROMIXS (Panjkovich and Svergun, 2018).

Further processing and modeling were done using ATSAS 3.0 (Franke et al., 2017). Scattering curves were analyzed and particle dimensions determined using PRIMUS (Konarev et al., 2003) and GNOM (Svergun, 1992), and initial particle shape determination was performed using BODIES and AMBIMETER (Konarev et al., 2003; Petoukhov and Svergun, 2015). Chain-like models were generated using GASBOR (Svergun et al., 2001). In combination with the crystal structure, hybrid modeling was performed using CORAL (Petoukhov et al., 2012). CRYSOL (Svergun et al., 1995) was used to evaluate the fits of crystal structures to experimental data. SUPCOMB was used to superimpose SAXS models and crystal structures (Kozin and Svergun, 2001).

Low-resolution electron density reconstructions were calculated using DENSS (Grant, 2018). The electron density maps were calculated 20 times and averaged using EMAN2 (Tang et al., 2007). SAXS data and models were deposited at the SASBDB (Supplementary Table S1).

### Multi-Angle Light Scattering

Protein molecular mass and heterogeneity were determined by multi-angle light scattering (MALS) using a miniDAWN TREOS II detector (Wyatt Technologies), coupled to a Shimadzu Prominence HPLC system with RID-20A (RI) and SPD-M30A (diode array) detectors. SEC to separate oligomeric species was performed using Superdex 75 10/300 GL or Superdex 200 15/150 GL increase columns (GE Healthcare) in SEC buffer. The protein concentration was 1–10 mg/ml, and the injected protein amount 15–150 µg. Data processing, baseline reduction, and molecular weight calculation were done in ASTRA 7 (Wyatt Technologies).

### Thermal Denaturation Assays

GDAP1∆319-358 and GDAP1∆295-358 in SEC buffer were titrated with HA (final DMSO concentration 2% (v/v)) in a 96-well PCR plate. After adding SYPRO Orange fluorescent dye, the plate was sealed with an optical PCR plate sheet, and thermal denaturation was analyzed by differential scanning fluorimetry (DSF) in an Applied Biosystems 7500 device. Melting curves were analyzed with GraphPad Prism.

### Label-free Stability Assay

Thermal unfolding of wild-type and C88A GDAP1∆303-358 in SEC buffer was studied by nanoDSF using a Prometheus NT.48 instrument (NanoTemper). The fluorescence of tryptophan was excited at 280 nm and recorded at 330 and 350 nm. The samples were heated from +20 to +90 °C with a heating rate of 1 °C/min, and changes in the fluorescence ratio (F_350_/F_330_) were monitored to determine apparent melting temperatures (T_m_).

### Isothermal Titration Calorimetry (ITC)

The binding affinity of GDAP1∆319-358 and GDAP1∆295-358 toward HA and GDAP1∆295-358 toward GSH was measured using a MicroCal iTC200 calorimeter (GE Healthcare) in SEC buffer with and without 2% (v/v) DMSO for HA and GSH, respectively. The sample cell and injection syringe were filled with 50 μM GDAP1 and 500 μM HA or 8 mM GSH, respectively. The system was equilibrated to a stable baseline before initiating an automated titration. The injection volume was 2.5 μL with 15 injections for HA and 3 μL with 12 injections for GSH. Injections were repeated at 180-s intervals at +25 °C. The sample was stirred at 750 rpm. The data were analyzed with the one-site binding model in Origin (MicroCal) to obtain thermodynamic parameters.

### Biolayer Interferometry (BLI)

BLI measurements were performed in SEC buffer containing 0.005% Tween 20 and 2% DMSO, using an Octet RED instrument (FortéBio) at +25 °C. Biotinylated GDAP1∆319-358 was loaded onto Super Streptavidin (SSA) biosensors (FortéBio) and quenched with 250 µL of 10 μg/ml biocytin. The association of GDAP1∆319-358 with HA at a series of concentrations was measured for 180 s. The dissociation was performed by washing the biosensors with binding buffer for 180 s. A reference measurement without biotinylated protein was subtracted from all curves. Data were analyzed using Data Analysis 11.0 (FortéBio).

### GST Activity Assay

Spectrophotometric activity measurements were done using the generic GST substrate analogs 1-chloro-2,4-dinitrobenzene (CDNB), 4-nitrobenzyl chloride (pNBC), and 1,2-epoxy-3-(*p*-nitrophenoxy)propane (EPNP) together with every GDAP1 construct. Absorbance was followed at 360 nm for a 500 µL reaction at **+**25 °C for 5 min, with a Jasco V-730 UV-VIS spectrophotometer (JASCO International Co. Ltd., Tokyo, Japan) in a 1-mm quartz cuvette (Hellma Analytics). Substrate concentrations in the assays were 1 mM (CDNB), 0.25 mM (pNBC), and 0.3 mM (EPNP) in 100 mM potassium phosphate buffer, pH 6.5. The GSH concentration was between 1–5 mM, and GDAP1 amount was 50 µg. As a positive control, 0.5 µg of recombinant *S. japonicum* GST (*Sj*GST)-TEV fusion protein was used. Data were analyzed using Jasco Spectral Analysis software. All measurements were done in triplicate.

### Glutathione-Sepharose Binding Assay

100 µL aliquots of GSH-sepharose 4B (GE Healthcare) matrix slurry were washed twice with deionized water. The matrices were collected in between washes by centrifuging at 300 *g* for 5 min at +4 °C. The matrices were equilibrated with 20 mM HEPES, 150 mM NaCl, pH 7.5, collected as above, and supernatants discarded. 40 µL of either 70 µM GDAP1Δ319-358 or 70 µM GDAP1Δ295-358 in 20 mM HEPES, 150 mM NaCl, pH 7.5 were added to the matrices and allowed to bind for 4 h at +4 °C. The matrices were centrifuged as above, and the supernatants (flow through fractions) were sampled for SDS-PAGE. The matrices were washed three times with 250 µL of 20 mM HEPES, 150 mM NaCl, pH 7.5, and after each wash, the matrices were collected as above and the supernatants sampled for SDS-PAGE. Bound proteins were eluted with 40 µL of 12 mM HEPES, 90 mM NaCl, 20 mM GSH, pH 7.5 for 15 min at +4°C, collected as above, and supernatants were sampled for SDS-PAGE.

## Results

### Identification of a Ligand Affecting GDAP1 Stability

**FIGURE 1:**
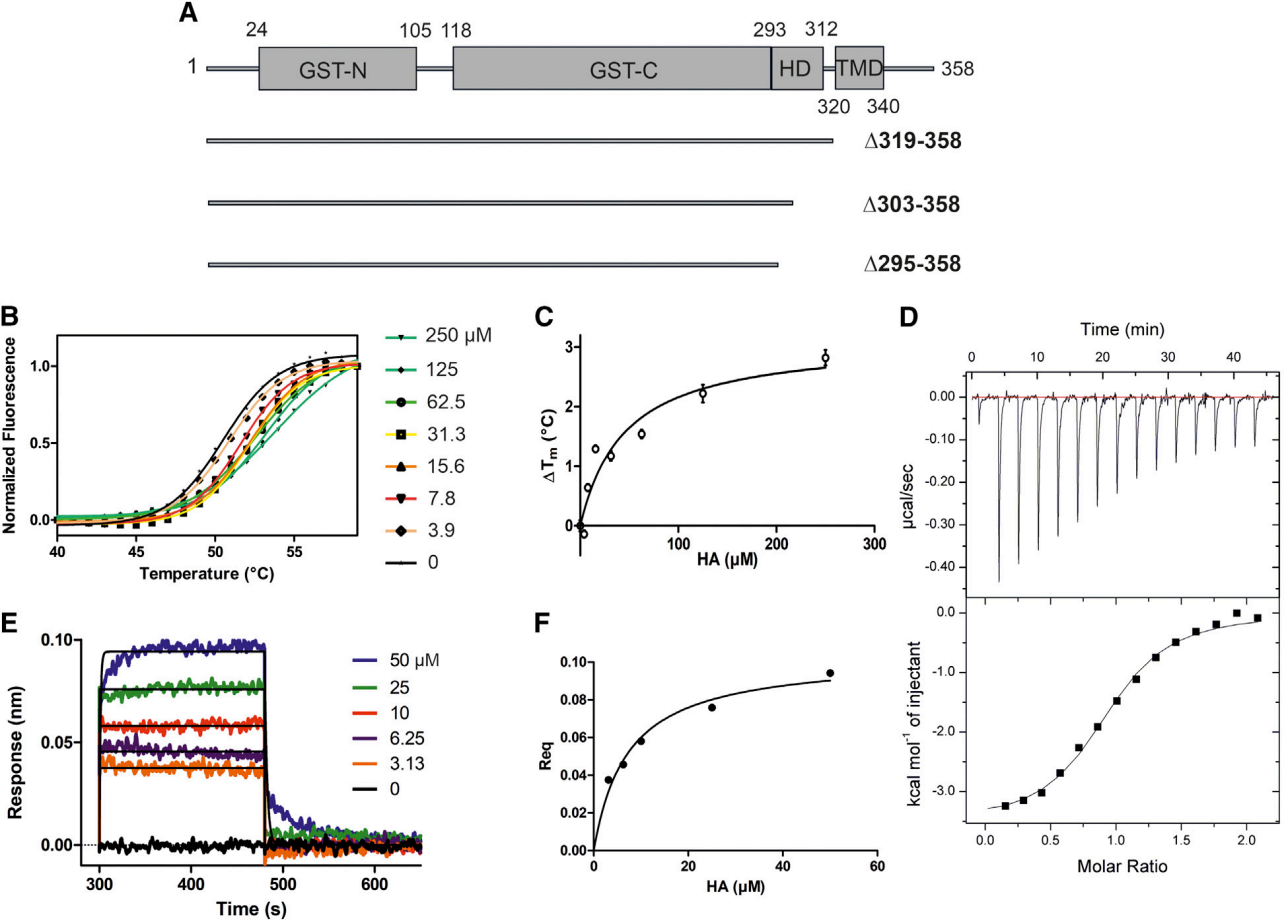
HA binding stabilizes GDAP1∆319-358. **(A)** Schematic of the full-length GDAP1 and three different deletion constructs used in this study. **(B)** Thermal unfolding data. **(C)** T_m_ shifts of GDAP1∆319-358 upon HA titration. **(D)** ITC binding curve of HA binding to GDAP1∆319-358. **(E)** BLI response of streptavidin-coated sensors derivatized with biotinylated GDAP1∆319-358 and exposed to increasing concentrations of HA. **(F)** Steady-state analysis of the BLI response vs. HA concentration.

We used the constructs GDAP1∆295-358, GDAP1∆303-358, and GDAP1∆319-358 to get detailed insights into human GDAP1 structure and potential functions (Figure 1A). Notably, the GDAP1-specific insertion (α-loop) was present in all constructs, in contrast to a recently reported mouse GDAP1 structure (Googins et al., 2020).

To search for ligands of GDAP1, compound library screening was performed using GDAP1∆319-358 and GDAP1∆295-358. Among ∼300 compounds in a metabolite library, HA showed an effect on GDAP1 stability, increasing its T_m_ by ∼3 °C. Due to its limited solubility, HA was titrated up to 250 μM, and a concentration-dependent T_m_ shift was observed (Figure 1B, C, and Supplementary Figures S1A, B, C). The binding affinity of HA to GDAP1∆295-358 and GDAP1∆319-358 was determined using ITC and BLI (Figure 1D, E, F, and Supplementary Figure S1D). The K_d_ values determined by ITC and BLI are in the same range, whereas a higher K_d_ is detected using DSF for GDAP1∆319-358 (Figure 1, Table 2). This could be due to an indirect effect from the fluorescent dye. Taken together, DSF, ITC, and BLI all show that HA binds to the GDAP1 core domain and stabilizes its structure.

**TABLE 2:** Binding affinities (K_d_, µM) of HA to GDAP1 using different methods.

Method	GDAP1∆319-358	GDAP1∆295-358
DSF	45.4 ± 18.3	9.7 ± 3.0
ITC	3.7 ± 0.2	2.0 ± 0.8
BLI	7.2 ± 1.5	Not determined

### GDAP1 Forms Dimers in Solution and HA Binding Affects Protein Oligomerization

**FIGURE 2:**
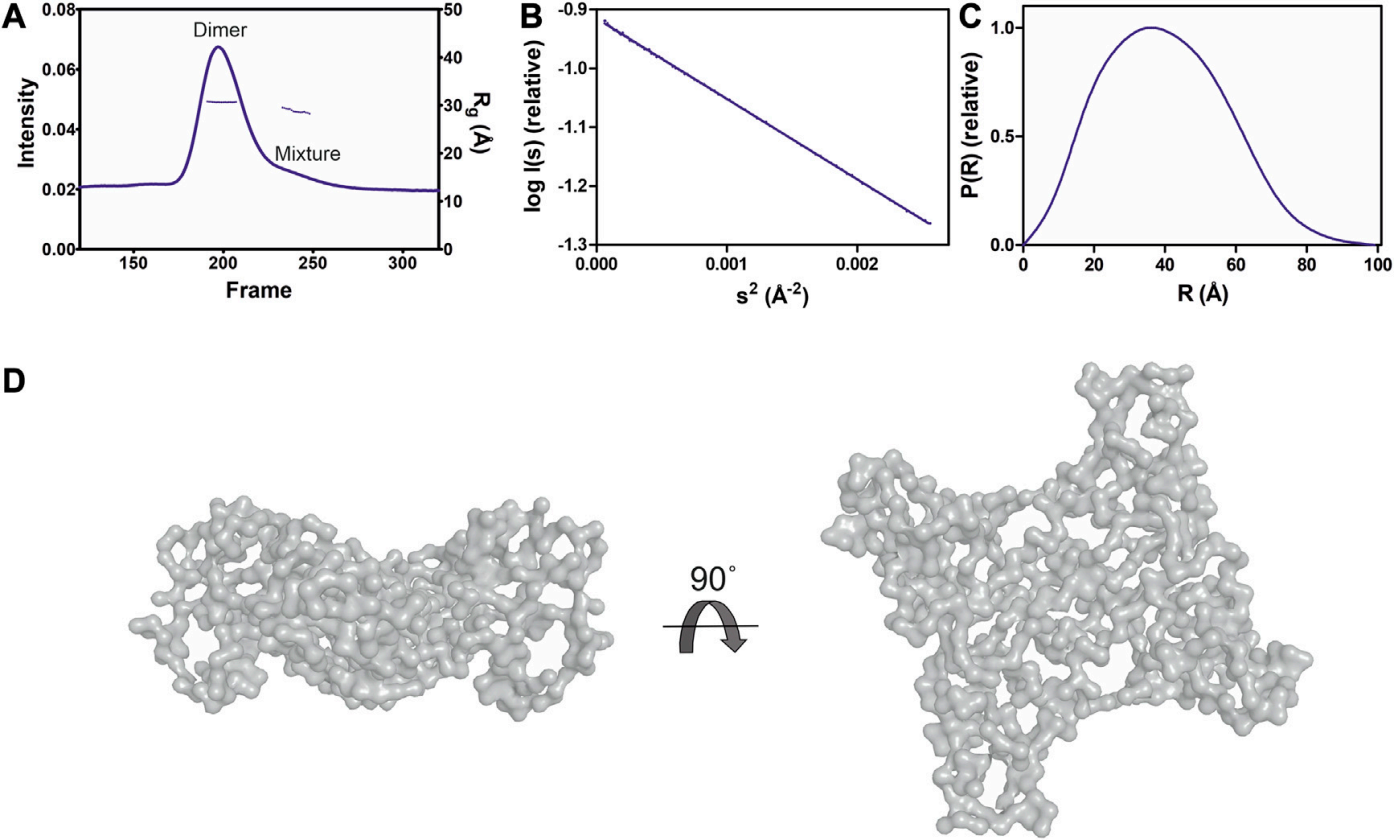
SAXS analysis of GDAP1∆303-358. **(A)** SEC-SAXS elution profile. R_g_ trace for the dimer and mixture of dimer/monomer peaks is also plotted. **(B)** Guinier analysis of the dimer data. **(C)** Distance distribution function for dimer. **(D)** Two different views of the *ab initio* chain-like model of dimeric GDAP1∆303-358.

GDAP1∆295-358 and GDAP1∆303-358 were subjected to synchrotron SEC-SAXS to investigate their oligomeric states and conformation. The separation between dimer and monomer peaks is best for GDAP1∆295-358 (Figure 2A and Supplementary Figure S2A). The linear fit in the Guinier region, the Porod volume, and the distance distribution function indicate monodispersity in the dimer peak of both constructs and the monomer peak of GDAP1∆295-358, but the small second peak of GDAP1∆303-358 appears to be a mixture of dimer and monomer. Clear separation of the monomer and dimer peaks enabled detailed analyses (Table 3, Figure 2B, C and Supplementary Figures S2B, C, D), and throughout this study, 3D modeling was only carried out for monodisperse, well-separated peaks. Figure 2D shows a chain-like *ab initio* dimer model of GDAP1∆303-358.

**TABLE 3:** SAXS structural parameters of wild-type and mutant GDAP1∆303-358 and GDAP1∆295-358.

	GDAP1∆303-358					GDAP1∆295-358	
Beamline	SWING/SOLEIL					P12/DESY	
Structural parameters	Wt dimer	Y29F	C88A main peak	C88A monomer	Y29E/C88A	Wt dimer	Wt monomer
R_g_ (Å) from P(r)	30.6 ± 0.12	30.4 ± 0.12	27.7 ± 0.11	26.0 ± 0.08	24.9 ± 0.10	31.2 ± 0.04	26.9 ± 0.05
R_g_ (Å) from Guinier plot	30.7 ± 0.03	30.4 ± 0.04	27.3 ± 0.04	25.6 ± 0.09	24.5 ± 0.04	31.3 ± 0.1	26.5 ± 0.1
D_max_ (Å)	99	98.7	93.2	85.1	86.7	101.7	92.3
Porod volume estimate, V_p_ (Å^3^)	105,750	101,874	71,901	63,520	58,695	108,944	71,457
Molecular weight determination (kDa)							
From Porod volume	76.2	74.2	50.4	42.3	34.6	84.0	53.7
From consensus Bayesian assessment	72.4	68.8	47.7	42.9	35.4	76.4	47.7
From V_C_	71.7	70.3	46.5	40.3	33.5	77.0	47.5
Ambimeter score	1.799	1.845	1.908	1.908	1.079		
Calculated monomeric MW from sequence	35.1					34.2	

To assess particle shape ambiguity, the scattering curves were analyzed using AMBIMETER and BODIES without restraints (Table 3). The slight score value variation suggests that both species are likely homogeneous and monodisperse, which agrees with the distance distribution functions.

To examine the concentration dependence of GDAP1 oligomerization, we tested two different concentrations for each construct using SEC. At lower concentrations, SEC data show two peaks corresponding to dimers and monomers, whereas broad peaks are observed at higher concentrations, implying a dimer/monomer equilibrium (Supplementary Figure S3). The main peak of GDAP1∆319-358 is a mixture of dimer and monomer and could only be separated at a very low concentration (Supplementary Figure S3B). Under non-reducing conditions, the protein adopts both dimeric and monomeric forms (Supplementary Figure S3D). Dimers are not detected under reducing conditions (Supplementary Figure S3D), indicating an inter-subunit disulfide bond involved in dimerization. These results are consistent with earlier observations that the endogenous GDAP1 dimer disappears in the presence of dithiothreitol (DTT) (Pedrola et al., 2005). Monomeric and dimeric GDAP1 can nevertheless be present in solution in dynamic equilibrium, as the dimer seems to form transiently and is dependent on the redox state.

**FIGURE 3:**
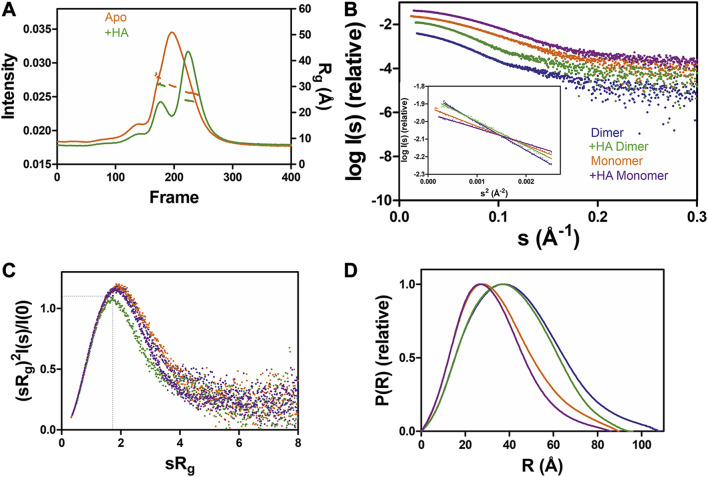
SAXS analysis of GDAP1∆319-358 in the absence and presence of HA. **(A)** SEC-SAXS elution profiles and R_g_ plot of SAXS frames for dimer and monomer peaks of the protein. **(B)** Experimental scattering data (log(I_s_) vs. s) and Guinier analysis (inset). **(C)** R_g_ normalized Kratky plots, the dashed lines representing the maximum value of a standard globular protein. **(D)** Distance distributions *p(r)* plots of ligand-free GDAP1 dimer (blue) and monomer (orange), ligand-bound GDAP1 dimer (green) and monomer (purple).

To test the effect of HA on GDAP1 oligomerization, we performed SEC-SAXS using GDAP1∆295-358 and GDAP1∆319-358. The elution profile of the protein-HA complex shows a higher monomer fraction than apo GDAP1 (Figure 3A and Supplementary Figure S4A). The apo GDAP1∆319–358 has a broad peak containing both dimer and monomer, whereas the complex elutes as two well-separated peaks (Figure 3A, B and Supplementary Figures S4A, B). Hence, HA binding allowed us to analyze monomeric and dimeric GDAP1 separately by SEC-SAXS.

In a dimensionless Kratky plot (Durand et al., 2010; Rambo and Tainer, 2011), folded globular proteins show a bell-shaped curve reaching its apex of 1.1 when sR_g_ = √3 , and multidomain proteins connected by linkers with a compact overall conformation have a bell-shaped curve, which is asymmetrically stretched (Kikhney and Svergun, 2015). The higher the sR_g_ value at the apex of the curve, the greater the flexibility and disorder of the protein (Durand et al., 2010). Dimensionless Kratky plots suggest that apo GDAP1 is less compact than HA-bound GDAP1 (Figure 3C and Supplementary Figure S4C), and distance distributions, as well as R_g_, indicate compaction of GDAP1 upon ligand binding for both monomeric and dimeric GDAP1 (Figure 3D, and Supplementary Figure S4D, Table 4). Hence, the stabilization of the GDAP1 structure is accompanied by a more compact 3D structure.

**TABLE 4:** SAXS structural parameters of GDAP1∆319-358 and GDAP1∆295-358 in the absence or presence of HA.

Structural parameters	GDAP1∆319-358				GDAP1∆295-358			
	SWING/SOLEIL							
	Apo dimer	Apo monomer	+HA dimer	+HA monomer	Apo dimer	Apo monomer	+HA dimer	+HA monomer
R_g_ (Å) from P(r)	33.5 ± 0.01	27.3 ± 0.01	31.2 ± 0.01	25.1 ± 0.01	32.2 ± 0.01	29.3 ± 0.01	30.9 ± 0.01	26.1 ± 0.01
R_g_ (Å) from Guinier plot	34.7 ± 0.22	27.1 ± 0.08	30.9 ± 0.14	24.6 ± 0.05	32.4 ± 0.07	29.1 ± 0.09	30.7 ± 0.08	25.3 ± 0.09
D_max_ (Å)	107.9	89.6	96	86	100.6	93.5	92.1	88.6
Porod volume estimate, V_p_ (Å^3^)	129,499	69,390	120,949	64,543	117,837	78,347	112,145	63,005
DAMMIN model volume	142,660	84,395	136,260	80,269	132,120	98,337	124,560	80,269
χ^2^ against raw data for GASBOR models	1.29	1.34	1.11	1.38	1.22	1.19	1.36	1.20
Molecular weight determination (kDa)								
From Porod volume	97.6	46.3	85.3	36.9	83.7	57.8	77.6	40.2
From DAMMIN model volume	71.3	42.2	68.1	40.1	66.1	49.2	62.3	40.1
From consensus Bayesian assessment	94.2	46.7	85.6	39.4	80.8	58.2	74.3	40.2
From V_C_	89.9	44.4	81	37.5	77.3	53.5	73	40.8
Calculated monomeric MW from sequence	36.9				34.2			

### Crystal Structures of Apo and Liganded GDAP1 Reveal Structural Relations to the GST Family but Suggest Lack of GST Activity

**FIGURE 4:**
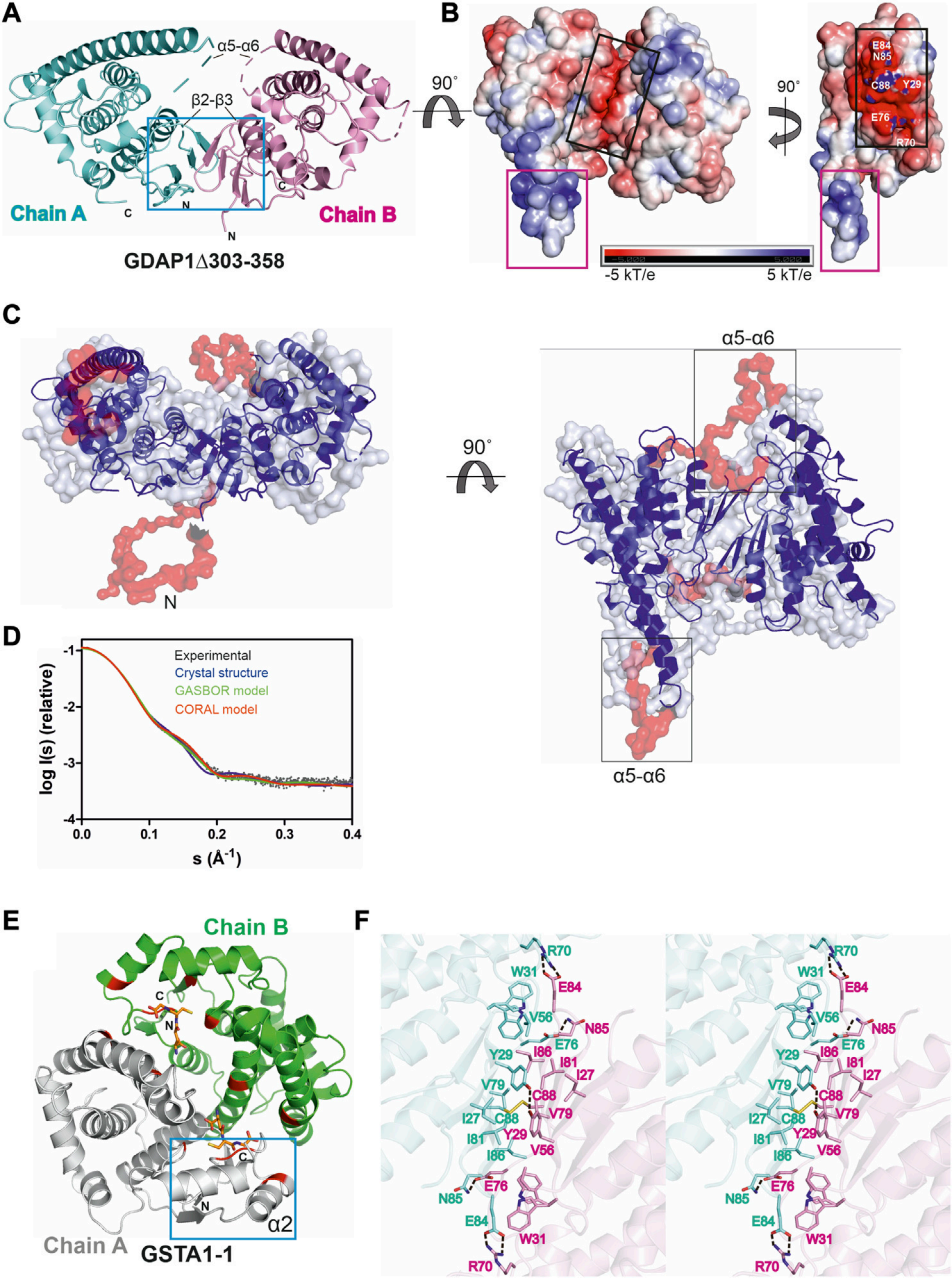
Crystal structure of the complete GDAP1 core domain and the dimer interface of GDAP1 compared to GSTA1-1. The orientation of GDAP1 chain A (cyan) and GSTA1-1 chain A (grey) are the same. **(A)** Overall structure of the dimeric GDAP1∆303-358. Chain A and chain B are shown in cyan and pink, respectively. The dashed lines indicate loops not defined by electron density. The dimerization interface is highlighted with a blue box. **(B)** Electrostatic surface potential of GDAP1∆303-358 is presented. The rotation shows only chain A. Note the strong positive potential of the long helix α6 (magenta box) and the negative potential of the dimer interface (black box). **(C)** Two different views of the *ab initio* chain-like model of dimeric GDAP1∆303-358 (transparent surface) superimposed with the GDAP1 crystal structure (blue) and the hybrid model. The built regions not present in the crystal structure are shown in red. **(D)** Experimental scattering curve of GDAP1∆303-358 dimer (black) overlaid with the theoretical scattering curve calculated from GDAP1∆303-358 structure (blue, χ^2^ = 17.1), GASBOR model (green, χ^2^ = 38.2) and CORAL model (red, χ^2^ = 4.7) using CRYSOL. **(E)** Dimeric GSTA1-1 (Grahn et al., 2006). GSH is shown as orange sticks. The catalytic residues are shown in red. Blue box is the GST-N region corresponding to GDAP1 dimer interface. Chain A and chain B are shown in grey and green, respectively. **(F)** Stereo view of the dimer interface showing interacting residues. The disulfide bond is shown, and hydrogen bonds are indicated by dashed lines.

We determined the crystal structure of human GDAP1∆303-358 at 2.8 Å resolution and its complex with HA at 2.2 Å resolution. Notably, this complete GDAP1 core domain, containing the GDAP1-specific ⍺-loop insertion, assembles as a homodimer (Table 1, Figure 4A). This is in contrast to the recently published structure of the mouse GDAP1 core domain, in which the GDAP1-specific insertion had been deliberately deleted (Googins et al., 2020).

Similarly to other GST family members, each GDAP1 monomer includes an N-terminal thioredoxin-like domain and a C-terminal α-helical domain. The GST-N domain has four β strands, forming a β sheet and two α helices with the topology β1-α1-β2-β3-β4-α2 (Supplementary Figures S5A, C), whereas, in canonical GST (Kursula et al., 2005), an additional α helix between β2 and β3 is present, forming an overall topology β1-α1-β2-α2-β3-β4-α3 (Supplementary Figures S5B, D). The GST-C domain is composed of seven α helices, with a long α6 helix visible in one monomer of the dimer. In the other chain, this helix is shorter (Figure 4A, B), implying flexibility of the α6 helix and a local breakdown of non-crystallographic symmetry. The β2-β3 loop, residues Ser73-Val77, and the α5-α6 loop, Gln163-Glu183, do not display clear electron density, also indicating flexibility (Figure 4A) even though the α5-α6 loop region was predicted to contain an additional α helix (Googins et al., 2020). The electrostatic potential map reveals mainly a negative charge close to the dimer interface, whereas a strong positive charge is found on the exposed surface of the long helix α6 (Figure 4B).

The chain-like SAXS *ab initio* dimer model superimposes well with the crystal structure (Figure 4C). A hybrid model of GDAP1∆303-358 was generated based on the crystal structure, building the missing residues (Figure 4C). This hybrid model fits the experimental SAXS data better than the chain-like model or a theoretical scattering curve generated from the crystal structure (Figure 4D). Hence, the conformation of GDAP1 in solution closely resembles that in the crystal state, and a simple rebuilding of the missing segments reproduces the solution scattering curve.

To complement the SAXS analysis, electron density reconstructions were prepared using DENSS (Grant, 2018) from GDAP1∆303-358 SAXS data. According to the averaged DENSS electron density map (Supplementary Figure S6), the conformational difference between the two subunits of the dimer in the crystal seems to also exist in solution. The α5-α6 loop is visible in the map, supporting the rigid body model of the missing loops. Although the dimer interface is small, the dimer is stable in solution. The particle dimensions computed from the maps agree with the distance distribution functions (Supplementary Table S2).

In contrast to canonical GST dimer interface contacts between GST-N of one subunit and GST-C of the other, involving β4, α3, α4, and α5 (Figure 4E), the dimer interface of GDAP1 forms entirely between the GST-N domains (Figure 4A, F). The interactions at the GDAP1 dimer interface include a disulfide bond between the Cys88 residues in strand β4 and a hydrogen bond between Tyr29 in strand β1 of each monomer (Figure 4F, Supplementary Figure S7A). Moreover, ion-dipole interactions between the Asn85 and Glu76 sidechains, as well as a salt bridge between Glu84 of one monomer and Arg70 of the other monomer, contribute to dimer formation (Figure 4F, Supplementary Figure S7A). Together with Tyr29, many residues, including Ile27, Trp31, Val56, Val79, Ile81, and Ile86, create a hydrophobic surface at the dimer interface (Figure 4F, Supplementary Figure S7A). The buried surface area of the GDAP1 dimer interface is 1,530 Å^2^, which is distinctively smaller than the 3,240 Å^2^ buried surface area of the canonical GST dimer. In this respect, it should be remembered that Cys88 lies in the middle of the GDAP1 interface and can lock the interface through disulfide formation. Thermodynamic parameters of the GDAP1 interface were compared to canonical GST (Krissinel 2015). The GDAP1 and canonical GST solvation energy gain upon interface formation was −10.4 kcal/mol (*p*-value = 0.0884) and −14.8 kcal/mol (*p*-value = 0.2393), respectively. The results suggest that the GDAP1 interface is part of the biological assembly rather than an artifact *in vitro*, despite having a small interface area. The above observations are consistent with the lack of GST activity in GDAP1, as the canonical dimerization mode generates the active site with sites for substrate binding. We could not detect any activity toward the conventional GST substrates CDNB, NBC, and EPNP, even at high protein concentration (Supplementary Table S3). Moreover, using a GSH sepharose binding assay and ITC, we confirmed that GDAP1 does not bind GSH (Supplementary Figure S8). Similarly, previous studies showed no GSH binding to GDAP1 using ITC (Googins et al., 2020) or no GSH-dependent activity (Shield et al., 2006).

**FIGURE 5:**
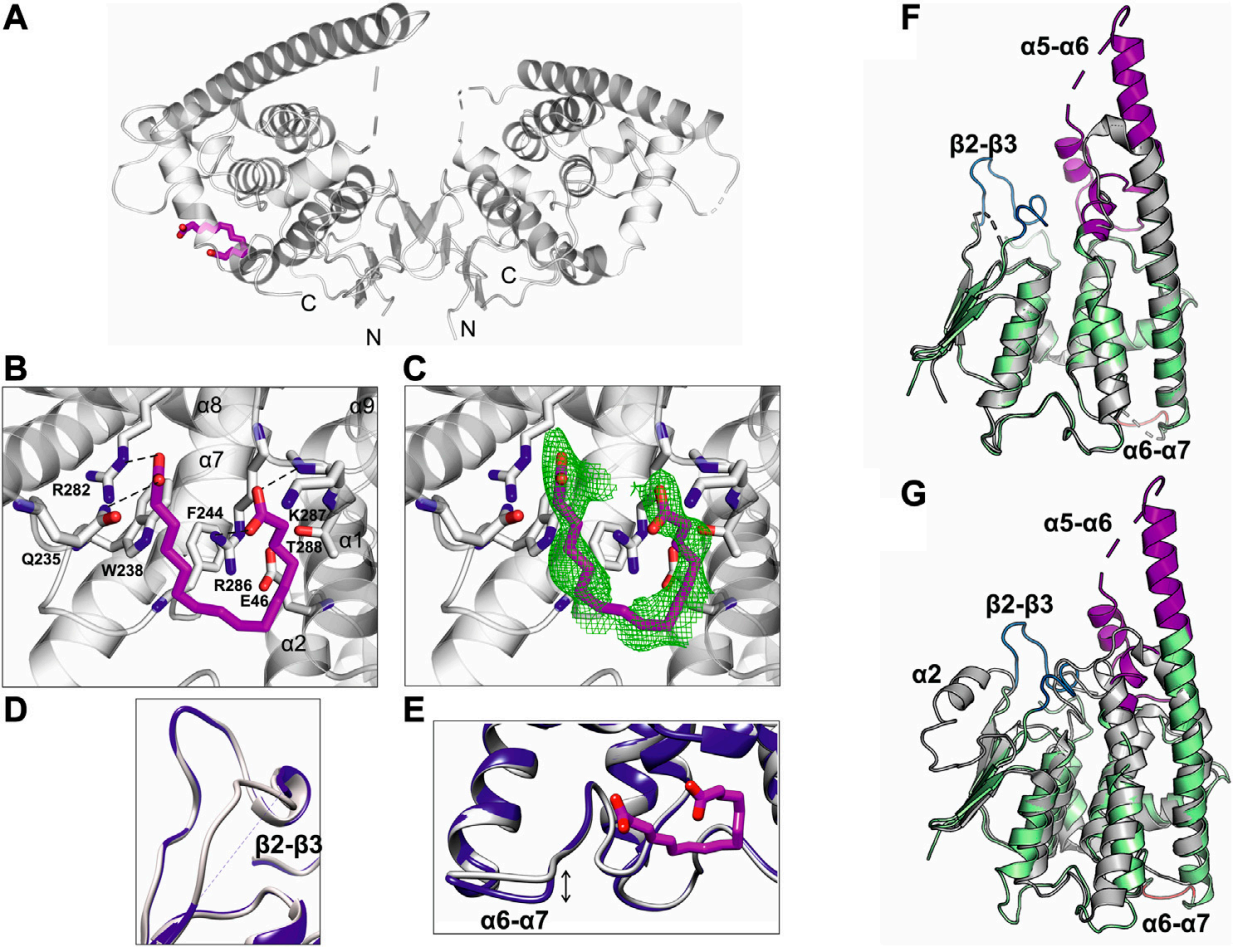
Crystal structure of GDAP1∆303-358 in complex with HA. **(A)** Overall structure of the GDAP1-HA complex. The dashed line indicates a loop not resolved in electron density. HA is shown in magenta. **(B)** HA interacting residues, hydrogen bonds are shown as dashed lines. **(C)** HA ligand of GDAP1 overlaid with Polder map (5σ, green). **(D)** Superposition the loop β2-β3 of apo GDAP1 (blue) and the HA complex (grey). The dashed line indicates a loop not resolved in electron density. **(E)** Superposition of the loop α6-α7 of apo GDAP1 (blue) and the HA complex (grey). HA is shown in magenta. **(F)** Superposition of human GDAP1 (green) and mouse GDAP1 (Googins et al., 2020) (grey). The dashed lines indicate loops not resolved in electron density. The mobile loops include β2-β3 (blue), α5-α6 (magenta), and α6-α7 (brown). **(G)** Superposition of human GDAP1 (green) and *Sj*GST (Kursula et al., 2005) (grey). The dashed lines indicate loops not resolved in electron density. The mobile loops include β2-β3 (blue), α5-α6 (magenta), and α6-α7 (brown).

The crystal structure of GDAP1 in complex with HA (Table 1) reveals the ligand-binding site. HA binds to a pocket in the C-terminal domain formed by helices α1, α8, and α9 and their connecting loops (Figure 5A). The side chains of Arg282 and Gln235, together with Lys287 and Arg286, make hydrogen bonds and salt bridges to the carboxyl groups of HA, whereas the alkyl moiety forms van der Waals interactions with residues lining the pocket, including Trp238, Phe244, and Thr288 (Figure 5B, C, and Supplementary Figure S7B). Superposition of human GDAP1 structures on truncated mouse GDAP1 (Googins et al., 2020) and *Sj*GST (Kursula et al., 2005) shows differences in loops β2-β3, α5-α6, and α6-α7 (Figure 5D, E, F, G). The loop β2-β3 of GDAP1 becomes more ordered in the presence of the ligand, whereas no electron density is present in this region in the apo structure and the mouse GDAP1 (Figure 5D). The loop β2-β3 contains the α2 helix in the canonical *Sj*GST structure (Figure 5G and Supplementary Figure S5). The α5-α6 loop in human GDAP1 is a unique structure compared to the truncated mouse GDAP1 and *Sj*GST; this segment corresponds to the GDAP1-specific long insertion (Figure 5F, G). The α6-α7 loop shift makes the structure more compact in the presence of the ligand (Figure 5E). This observation is consistent with SAXS data, which showed more compact conformations in the presence of HA (Supplementary Figure S9).

To summarize, although the GDAP1 core domain and canonical GSTs share a similar monomer fold, the crystal structure of GDAP1 reveals a novel dimer interface. The lack of GST activity and GSH binding confirm that GDAP1 has a unique structure and function compared to the rest of the GST family. HA plays a role as an allosteric modulator of oligomerization, flexibility, and stability of GDAP1, at least *in vitro*.

### Identification of Key Residues for GDAP1 Dimerization

**FIGURE 6:**
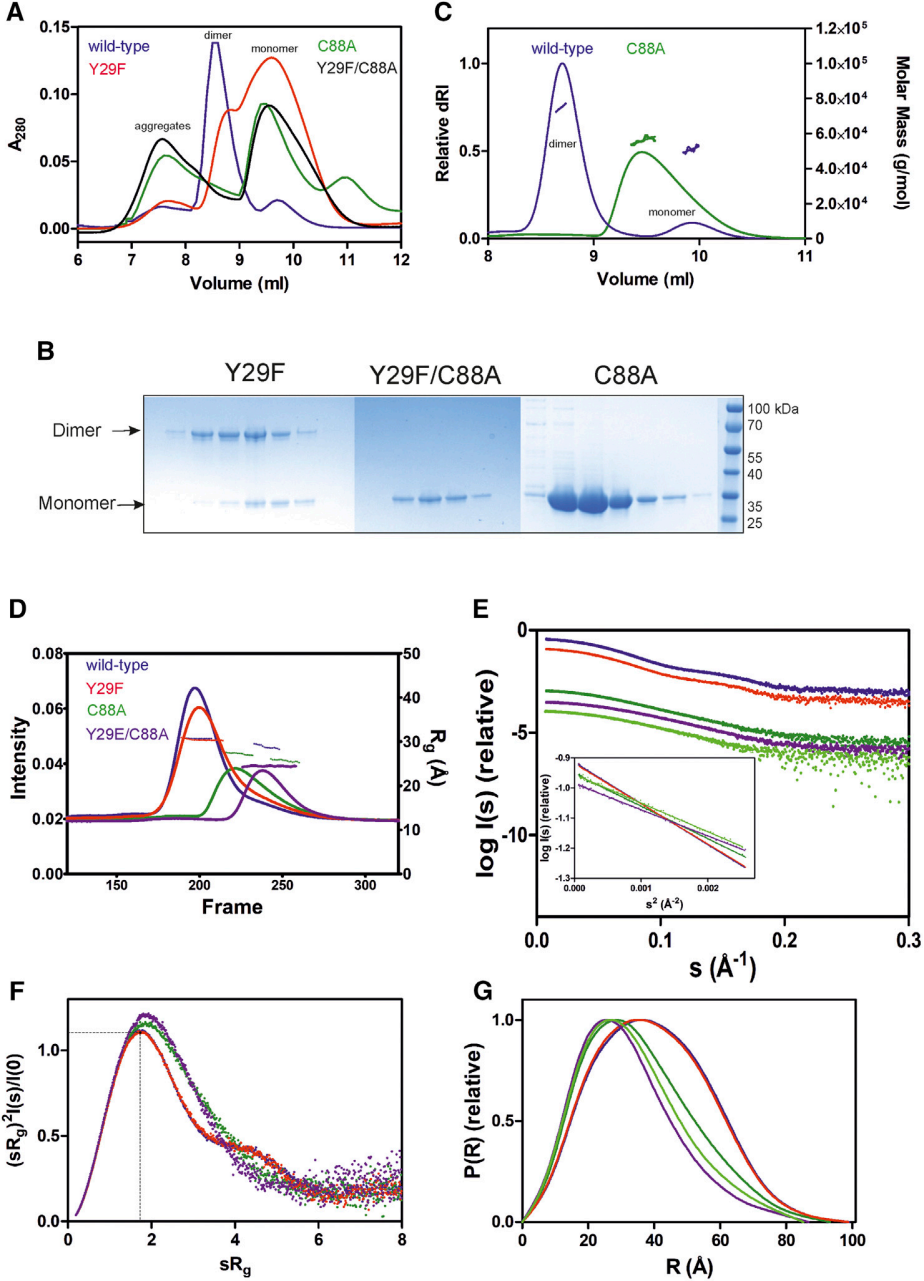
The oligomerization of wild-type and mutant GDAP1∆303-358 using SEC, SEC-MALS and SEC-SAXS. **(A)** SEC elution profile of wild-type (purple), C88A (green), Y29F (dark orange), and Y29F/C88A (black) using column S75 increase 10/300 GL. **(B)** Non-reducing SDS-PAGE gel of mutants SEC elution. **(C)** SEC-MALS analysis of wild-type (purple) and C88A (green) using column S75 increase 10/300 GL. **(D)** SEC-SAXS elution profiles and R_g_ plots. **(E)** Experimental scattering data (log(I_s_) vs s) and linear fits in the Guinier regions (inset). The main and the second half of C88A peak are shown in dark and light green, respectively. **(F)** R_g_ normalized Kratky plots, the dashed lines representing the maximum value of a standard globular protein. **(G)** Distance distributions *p(r)* plots of wild-type GDAP1∆303-358 dimer (blue) and mutants Y29F (red), Y29E/C88A (purple), C88A main peak (dark green), and the second half (light green).

The crystal structure of GDAP1 reveals Cys88 and Tyr29 as central residues for dimer formation (Figure 4F). To confirm their essential role at the dimer interface, mutations were generated, including C88A, Y29F, Y29F/C88A, and Y29E/C88A, and oligomerization was investigated using SEC, SEC-MALS, and SEC-SAXS. Comparison of SEC elution profiles shows that the Y29F mutant retains a small amount of dimer, whereas the C88A and Y29F/C88A mutations significantly inhibit dimer formation (Figure 6A). In non-reducing SDS-PAGE, a dimer band is present for Y29F but absent for C88A and Y29F/C88A (Figure 6B). According to SEC-MALS, the main peak for wild-type GDAP1 is a dimer, whereas C88A GDAP1 is a mixture of dimer and monomer with an apparent mass of 55.3 ± 4.8 kDa, similar to the second peak of the wild-type protein (Figure 6C). To examine the stability of wild-type GDAP1 and the C88A mutant, we used nanoDSF, a label-free fluorimetric technique that can determine the thermostability of proteins by following changes in their intrinsic fluorescence. The T_m_ for wild-type GDAP1 and C88A were +62.1 ± 0.23 °C and +57.4 ± 0.01 °C, respectively, which probably reflects the larger dimer fraction of wild-type GDAP1. Taken together, Cys88 is important for GDAP1 dimerization and stability.

To confirm the role of Tyr29 and Cys88 for GDAP1 dimerization, oligomerization of the mutants was studied using SEC-SAXS. Wild-type GDAP1 and the Y29F mutant eluted as a dimeric form, whereas C88A and Y29E/C88A eluted later (Figure 6D). In SAXS, Y29F at high concentration (10 mg/ml) shows a more substantial dimer peak in comparison to SEC data (Figure 6A–5 mg/ml), implying that the hydrogen bond between Tyr29 residues is involved in dimerization, but not strictly required. The scattering curves and Guinier fits confirm sample monodispersity (Figure 6E).

According to the dimensionless Kratky plot, dimeric wild-type GDAP1∆303-358 is compact, whereas the monomeric form observed for mutant proteins is less compact (Figure 6F). The D_max_ of the monomeric C88A and Y29E/C88A variants is shorter compared to dimeric wild-type GDAP1 (Figure 6G and Table 3). The main peak of C88A has a larger molecular weight compared to Y29E/C88A (Table 3), suggesting that this peak is a mixture of dimer and monomer. In contrast, the second part of the peak represents a monomer and shows a molecular weight and distance distribution similar to Y29E/C88A (Figure 6G, Table 3). The *ab initio* model of Y29E/C88A superimposes well with the crystal structure of GDAP1 chain A (Supplementary Figure S10A). Particle shape reconstruction was done for the Y29E/C88A mutant (Supplementary Figure S10B). According to its R_g_, it is monomeric. The *ab initio* map corresponds to a clearly non-spherical shape, indicating that the GDAP1 monomer exists in an extended conformation in solution.

For further insight into the structure of monomeric GDAP1, low-resolution electron density maps were reconstructed for the GDAP1∆303-358 wild-type and the Y29E/C88A mutant. The averaged Y29E/C88A map reveals a monomeric particle, in line with the *ab initio* model (Supplementary Figure S10B). The map reveals a shape similar to the monomeric mouse GDAP1 crystal structure (Googins et al., 2020).

Taken together, SEC, SEC-SAXS, and SEC-MALS data confirm that Cys88 plays an important role at the dimer interface. Tyr29 contributes with a regular hydrogen bond, a C-H…π bond to Ile81, and a number of van der Waals interactions. The mutation Y29E/C88A abolished the disulfide bond and disrupted the hydrophobic surface on the dimer interface, generating a monomeric form.

### GDAP1L1 Is Monomeric

**FIGURE 7:**
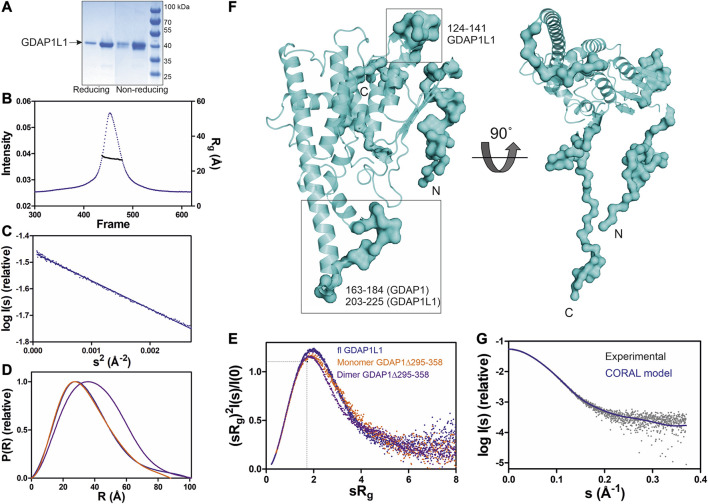
SAXS analysis of GDAP1L1. **(A)** Non reducing and reducing SDS-PAGE gel of GDAP1L1. **(B)** SEC-SAXS elution profile and R_g_ plot of SAXS frames. **(C)** Guinier analysis. **(D)** Distance distributions *p(r)* plots. **(E)** Dimensionless Kratky plots for GDAP1L1 (blue) and GDAP1 monomer (orange) and dimer (purple). The dashed lines represent the peak position for a standard globular protein. **(F)** CORAL model of GDAP1L1 based on GDAP1 crystal structure (cartoon), surface for the restored missing fragments. **(G)** Experimental scattering curve of GDAP1L1 (grey) overlaid with the CORAL model (blue, χ^2^ = 1.19) using CRYSOL.

GDAP1L1 is a paralogue of GDAP1 with 55% sequence identity (Supplementary Figure S11) and is mainly cytosolic (Niemann et al., 2014). As opposed to full-length GDAP1 (data not shown), full-length GDAP1L1 over-expressed in *E. coli* can be purified to homogeneity and is soluble (Figure 7A). Under both non-reducing and reducing conditions, GDAP1L1 migrates as a 44-kDa monomer on SDS-PAGE (Figure 7A). Mass spectrometry confirmed the protein band to be full-length GDAP1L1. Sequence alignments show that Cys88 and Glu84, involved in the dimer interface of GDAP1, are replaced by Ser109 and Asp105, respectively, in GDAP1L1 (Supplementary Figure S11). Coupled with the high sequence similarity, GDAP1L1 folds like GDAP1 but does not form dimers.

SEC-SAXS was used to study the oligomeric state of GDAP1L1, revealing an R_g_ of 27 Å (Figure 7B). In line with this, the GDAP1L1 molecular mass calculated from volume correlation is 43.7 kDa, and from Bayesian estimate 44.7 kDa. A linear Guinier fit indicates that GDAP1L1 is quantitatively monomeric (Figure 7C). The P(r) function of GDAP1L1 has a similar shape as monomeric GDAP1∆295-358, except for a long tail, leading to a D_max_ of 100 Å (Figure 7D). This tail implies that GDAP1L1 has disordered regions, most likely corresponding to the N terminus and the C-terminal HD and TMD. It thus seems that the single transmembrane domain does not make recombinant GDAP1L1 insoluble; this behavior is different from GDAP1 and could be related to the different oligomeric states. The dimensionless Kratky plot of GDAP1L1 shows an asymmetric bell-shaped curve (Figure 7E), indicating increased structural flexibility compared to the GDAP1 core domain. A SAXS-based hybrid model of full-length GDAP1L1 was generated based on the GDAP1 crystal structure and complemented with the missing loops and termini (Figure 7F). This hybrid model fits the experimental data (Figure 7G) and shows flexible regions in addition to the folded monomeric core domain (Figure 7F).

## Discussion

We carried out a detailed structural characterization of human GDAP1 containing the full GDAP1-specific insertion, containing the α5-α6 loop and the long α6 helix. The results indicate that GDAP1 forms a unique type of homodimer mediated by a hydrophobic surface and a disulfide bridge. Furthermore, a fatty acid ligand for GDAP1 was identified. Together with earlier data, our results provide important clues toward the structure and function of GDAP1 on the outer mitochondrial membrane and its involvement in neurodegenerative disease.

### GDAP1 is a Unique Member of the GST Family

Although the sequence identity is only ∼20%, the GDAP1 core domain shares a fold similar to canonical GST enzymes. However, GDAP1 has a unique mode of dimerization, and it lacks GST activity. The main differences constitute a missing α helix between β2 and β3 and the unique helices α5 and α6 with the connecting α5-α6 loop (Figure 4, 5, Supplementary Figure S5). Variations in these regions prevent GDAP1 from forming canonical GST dimers and interacting with typical GST substrates. Dimerization is critical for GST activity in all eight known GST classes (Mannervik et al., 1985; Meyer et al., 1991). Mutations at the GST dimer interface result in a stable, soluble, but inactive enzyme (Abdalla et al., 2002). The unique arrangement of the GDAP1 interface suggests a different function for GDAP1.

In α, µ, π, and *Sj*GST, a “lock-and-key” kind of hydrophobic interaction is established by wedging a hydrophobic side chain (Phe52, α; Phe56, µ; Phe47, π; Phe51, S*j*GST) from one monomer into a hydrophobic pocket on the second one, formed by five conserved residues on helices α4 and α5 (Ji et al., 1995). In GDAP1, the “key” Phe and “lock” residues are not conserved (Supplementary Figure S13).

The regions β2-α2-β3 and α4-α5 form the GSH binding site of GSTs, involving many interacting residues (Grahn et al., 2006), which are not conserved in GDAP1 (Supplementary Figures S12, 13). Googins *et al.* identified differences between the G-sites of GDAP1 and canonical GSTs, including limited sequence conservation in the α2 region (Googins et al., 2020). Contrary to predictions, we show that GDAP1 lacks helix α2. Apo and GSH-bound GSTA1-1 show a conformation of the α2 helix, which is completely different from the GDAP1 loop β2-β3 (Supplementary Figure S12). On the other hand, the catalytic Tyr9 residue of GSTA1-1 is conserved as Tyr29 in GDAP1, but Tyr29 points in another direction and makes central contacts at the GDAP1 dimer interface (Supplementary Figure S12). Hence, a similar fold makes GDAP1 a member of the GST enzyme family, but differences in the dimer interface and important residues for GSH binding and catalysis imply a unique function within the family.

### GDAP1 as a Target for CMT Mutations

**FIGURE 8:**
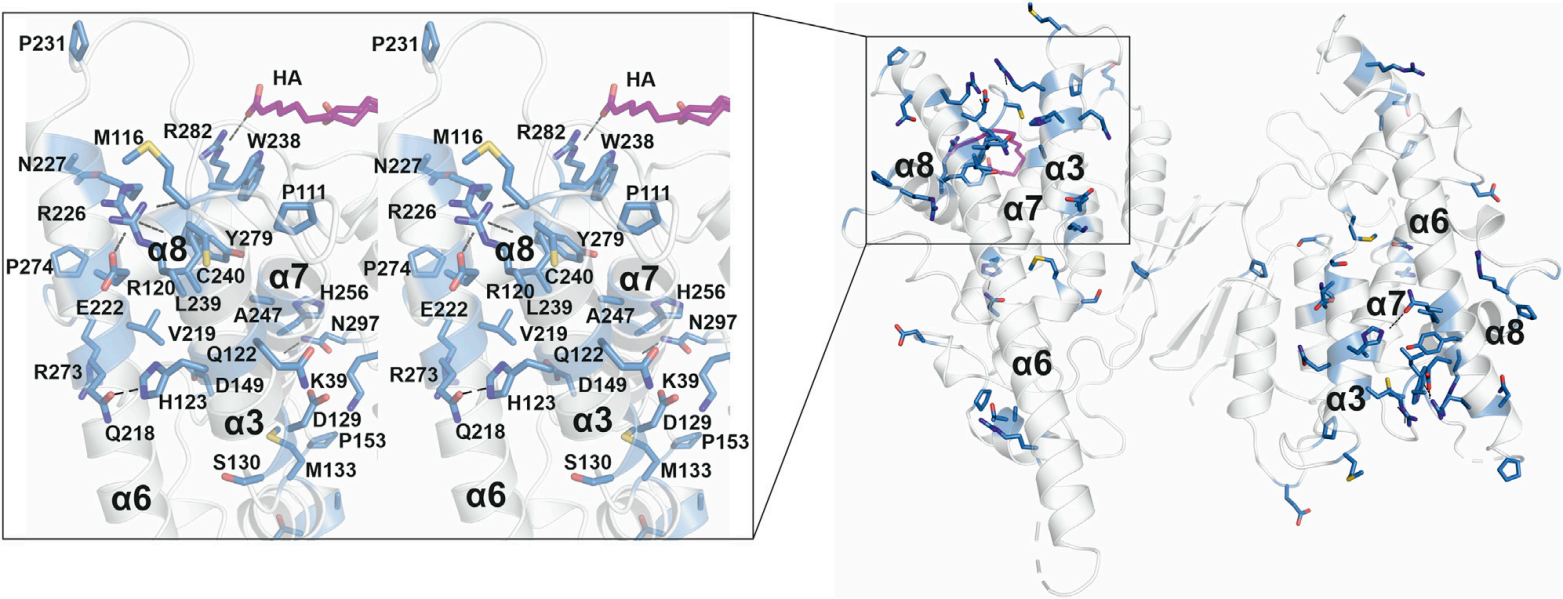
CMT-related residue cluster. Close-up stereo view of the CMT-related residue cluster (sticks) and HA binding site. Hydrogen bonds are shown as dashed lines. HA is shown in magenta.

A large number of CMT-related mutations in *GDAP1* have been identified. The most common *GDAP1* genotype in 99 Spanish patients was p.R120W (Sivera et al., 2017). R120W, H123R, A156G, and P274L were reported in European patients (Zimoń et al., 2011). Several mutations have been studied using neurons and Schwann cells or a yeast model (Estela et al., 2011; Zimoń et al., 2011; Rzepnikowska et al., 2020). The GDAP1 crystal structure now allows establishing a molecular basis for many of the known mutations in the human gene mutation database (http://www.hgmd.cf.ac.uk/ac). A CMT-related mutation cluster of GDAP1 mainly localizes on helices α3 and α6, and less on helices α7, α8, and their connecting loops (Figure 8). There are 46 published missense mutations involving 39 residues. The main cluster contains 27 residues that interact closely with each other in the crystal structure, including salt bridges, hydrogen bonds, and van der Waals interactions, forming a network of interactions (Figure 8). CMT mutations hindering these interactions could affect GDAP1 folding and stability, in addition to its interactions with other molecules. Interestingly, HA binds to GDAP1 in a pocket next to this cluster and forms a hydrogen bond with Arg282 (Figure 8). HA binding increases GDAP1 stability by inducing a conformational change of the loop α6-α7, which is involved in the mutation cluster. Thus, the CMT-related cluster and HA binding site may relate to the function and/or folding of GDAP1. GDAP1 is highly conserved between vertebrates but not fruit fly (Supplementary Figure S11). Crucial residues on the GDAP1 dimer interface, including Tyr29 and Cys88, and many CMT-related residues are conserved, suggesting a role in the structure and function of GDAP1. Further studies are needed to investigate GDAP1 function and its relation to CMT, and current structural data provide a strong basis for targeted experiments.

### GDAP1 and GDAP1L1 Comparison

As a paralogue of GDAP1, GDAP1L1 shares a high sequence identity and the same fold. However, in contrast to GDAP1, full-length GDAP1L1 is monomeric and soluble (Figure 7). GDAP1 and GDAP1L1 have many conserved residues at the GDAP1 dimer interface, except for the central residues Cys88 and Glu84 (Supplementary Figure S11). Gly83, a residue localized at the hydrophobic surface on the dimer interface, is replaced by Arg in GDAP1L1 (Supplementary Figure S11). G83R is a CMT-related mutation in an Italian family (Geroldi et al., 2007). GDAP1L1 might have one additional α helix between α2 and α3 (Supplementary Figure S11). A shorter C terminus could be linked to the observed solubility of full-length GDAP1L1 compared to GDAP1.

Due to the conserved HD and TMD (Supplementary Figure S11), GDAP1L1 can target mitochondria and compensate for GDAP1 deficiency (Wagner et al., 2009). Hence, it appears that the HD and TMD are essential for GDAP1/GDAP1L1 mitochondrial targeting, while the GST-N and GST-C domains play a role in another function.

### Functional Considerations

The unique α-loop of GDAP1 is involved in interactions with β-tubulin (Estela et al., 2011; Pla-Martín et al., 2013), indicating that GDAP1 may participate in the interaction between mitochondria and microtubules. The CMT-related cluster and the HA binding site highlight an important region of GDAP1. This region could be a binding pocket for a substrate or co-factor to catalyze a reaction if the protein functions as an enzyme. The region could also be a contact surface with other proteins, such as β-tubulin. It has been shown that the interactions between GDAP1 and β-tubulin were highly increased for the GDAP1 mutants at the CMT-related cluster and the long α6 helix, including R120Q, R120W, T157P, R161H, and R282C, pointing toward a gain-of-function mechanism that affects spindle formation (Estela et al., 2011). It was speculated that *via* interaction with GDAP1 and other fission proteins, microtubules could be important for the interaction between mitochondria and the cytoskeleton (Estela et al., 2011).

GDAP1 was reported to interact with Rab6B, a protein localized to the Golgi apparatus and distributed in Golgi and ER membranes (Opdam et al., 2000), and with caytaxin, a protein involved in mitochondrial transport (Pla-Martín et al., 2013). The interaction between these proteins may be important for the localization of mitochondria close to SOCE sites (González-Sánchez et al., 2017). GDAP1 mutations in the α-loop could perturb protein interactions, thus inhibiting SOCE activity or stimulating abnormal mitochondrial distribution (González-Sánchez et al., 2017).

GDAP1 is not only located in mitochondria, but also in mitochondria-associated membranes (MAMs), and it may play a role at the interface between mitochondria and the ER (Pla-Martín et al., 2013). GDAP1-linked CMT may be associated with abnormal distribution and movement of mitochondria along the cytoskeleton toward the ER and subplasmalemmal microdomains (Pla-Martín et al., 2013). The bidirectional movement of lipids between the ER and mitochondria may be mediated by interactions between MAM and mitochondria (Vance, 2014). Literature regarding HA clearly suggests a role in mitochondrial membrane permeability (Dubinin et al., 2013; Dubinin et al., 2014a; Dubinin et al., 2014b; Vedernikov et al., 2015). This aspect opens up potential new lines of research with respect to GDAP1, HA, and mitochondrial metabolism and permeability.

Fatty acids are a source of metabolic energy and function as building blocks for complex lipids. GDAP1 could be a fatty acid transport protein due to its localization on MAM and MOM and its fatty acid binding shown here. Moreover, since both GDAP1 and HA are linked to Ca^2+^ homeostasis, GDAP1 may regulate this metabolism through its binding to fatty acids.

Another aspect arising from these findings is the oligomeric state of GDAP1 *in vivo*. As shown by earlier studies from neuronal cell line protein extracts (Pedrola et al., 2005), GDAP1 seems to be expressed as a dimer, and our results show that the dimers are covalently bonded. Changes in the redox environment could easily alter this equilibrium. In cells, mutant variants of GDAP1 lead to depleted GSH levels, causing excess reactive oxygen species (ROS) stress, suggesting that GDAP1 may actively regulate GSH metabolism. These could affect mitochondrial membrane integrity and oxidative phosphorylation efficiency *via* an unknown mechanism (Niemann et al., 2009; Noack et al., 2012; Cassereau et al., 2020). Thus, the oligomerization of GDAP1 could be a regulated event induced by specific ROS-sensitive pathways.

### Insights into the Structure of Full-Length GDAP1

**FIGURE 9:**
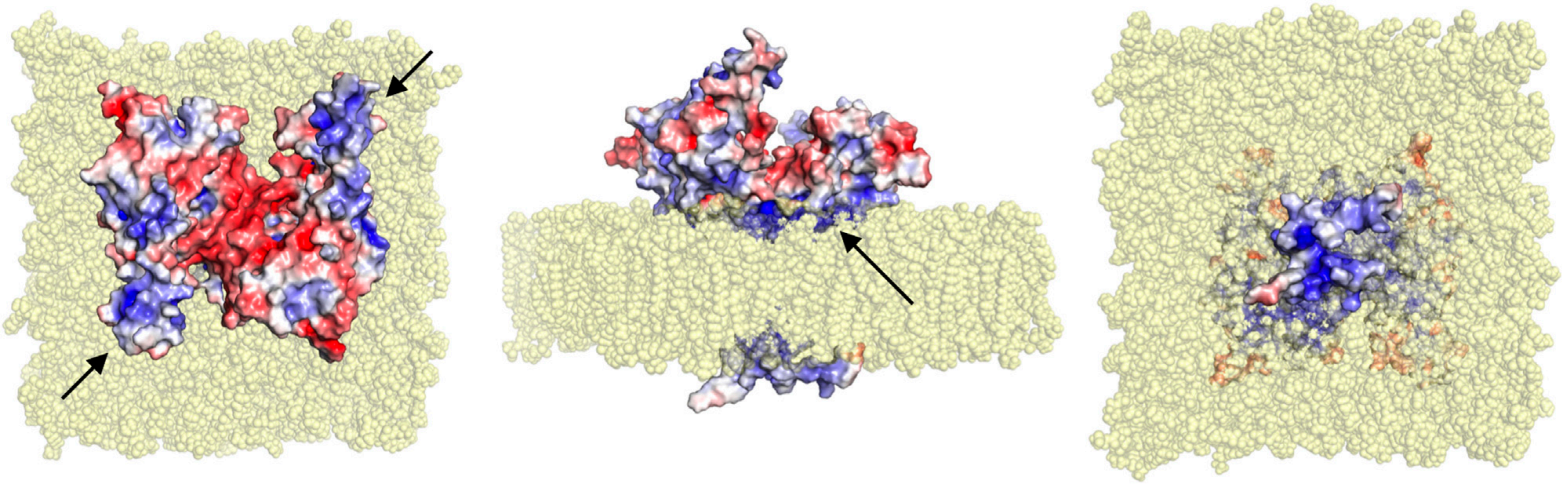
Model for full-length dimeric GDAP1 on a membrane bilayer. Full-length GDAP1, including modeled TMD and loops missing from the crystal structure, is shown as an electrostatic potential surface. In the top view (left), note how the cavity at the dimer interface has negative potential, while the long helices and the ⍺5-⍺6 loop (arrows) have positive charge. The side view (middle) shows a cavity in the dimer arrangement toward the cytosol (top) and a positively charged surface facing the membrane (arrow). The bottom view (right) indicates that the tail is very small and positively charged.

The crystal structure of the dimeric GDAP1 core domain lacks the HD and TMD, but full-length GDAP1 does form dimers in cells. Co-immunoprecipitation of full-length GDAP1 from HEK-293T cells confirmed that the protein formed homodimers (Huber et al., 2016). We built a model of dimeric full-length GDAP1 on a phospholipid membrane using the crystal structure of the complete human GDAP1 core domain (Figure 9). The transmembrane domain of GDAP1 contains a Gly zipper, a motif linked to the dimerization of transmembrane helices (Kim et al., 2005). The lipid fraction of MOM in mammals consists mainly of phosphatidylcholine, phosphatidylethanolamine, and phosphatidylinositol (Daum and Vance, 1997), with minor amounts of phosphatidylserine, cardiolipin, and phosphatidic acid. On both sides of the membrane, the model shows positively charged surfaces of the protein at the bilayer headgroup regions. The GDAP1-specific insertion has a strong positive potential and could be involved in molecular interactions, for example, with the cytoskeleton. The model can serve as a starting point for more detailed functional and computational studies in the future.

The fatty acid-binding site observed in the GDAP1 crystal structure faces the membrane-binding surface in the context of the modeled full-length dimer, suggesting that the observed ligand could mimic the lipid membrane surface. This, in turn, suggests that membrane binding could affect the conformation of the GDAP1 core domain, for example, *via* the incorporation of acidic lipid headgroups in the binding site. These questions can be answered when the structure of full-length GDAP1 on a MOM-like lipid membrane eventually becomes available.

### Concluding Remarks

GDAP1 is a member of the GST family linked to CMT; however, its function remains unclear at the molecular level. The crystal structure of the complete human GDAP1 core domain reveals a GST-like fold, with a previously unseen mode for dimerization. The monomer-dimer equilibrium could be further linked to redox phenomena in the cell, and the function of full-length GDAP1 on the MOM may be regulated by the oligomeric state. The GDAP1 structure and the discovery of the first GDAP1 ligand not only provide information to map the CMT-related residue cluster and the corresponding interactions in detail, but also provides a template conformation for further functional studies and structure-assisted ligand design. Further studies on GDAP1-linked CMT should use the human GDAP1 crystal structure as a reference framework to explain the effects of mutations at the molecular level.

## Data Availability

The datasets presented in this study can be found in online repositories. The names of the repository/repositories and accession number(s) can be found below: http://www.wwpdb.org/, 7ALM, 7AIA https://www.sasbdb.org, SASDJR8, SASDJS8, SASDJT8, SASDJU8, SASDJV8, SASDJW8, SASDJX8, SASDJY8, SASDJZ8, SASDJ29, SASDJ39.
