# Erratum: Structure of the Complete Dimeric Human GDAP1 Core Domain Provides Insights Into Ligand Binding and Clustering of Disease Mutations

**DOI:** 10.3389/fmolb.2022.889681

**Published:** 2022-03-15

**Authors:** Giang Thi Tuyet Nguyen, Aleksi Sutinen, Arne Raasakka, Gopinath Muruganandam, Remy Loris, Petri Kursula

**Affiliations:** ^1^ Faculty of Biochemistry and Molecular Medicine and Biocenter Oulu, University of Oulu, Oulu, Finland; ^2^ Department of Biomedicine, University of Bergen, Bergen, Norway; ^3^ VIB-VUB Center for Structural Biology, Vlaams Instituut voor Biotechnologie, Brussels, Belgium; ^4^ Department of Bioengineering Sciences, Structural Biology Brussels, Vrije Universiteit Brussel, Brussels, Belgium

**Keywords:** protein structure, ganglioside-induced differentiation-associated protein 1, Charcot-Marie-Tooth disease, oligomeric state, fatty acid, membrane protein

Due to a production error, there was an error in the affiliations published.

Authors Giang Thi Tuyet Nguyen and Aleksi Sutinen should only have the following affiliation: “Faculty of Biochemistry and Molecular Medicine and Biocenter Oulu, University of Oulu, Oulu, Finland”. Author Arne Raasakka should have the following affiliation: “Department of Biomedicine, University of Bergen, Bergen, Norway”.

The publisher apologizes for this mistake. The original version of this article has been updated.

