# Supplementary material for: Structure of the Complete Dimeric Human GDAP1 Core Domain Provides Insights into Ligand Binding and Clustering of Disease Mutations
Source: Front Mol Biosci. 2021 Jan 27;7:631232. doi: 10.3389/fmolb.2020.631232 (PMC7873046; doi:10.3389/fmolb.2020.631232)
Supplement: Supplementary file 1 [file datasheet1.pdf]

Supplementary Material

1 Supplementary Figures

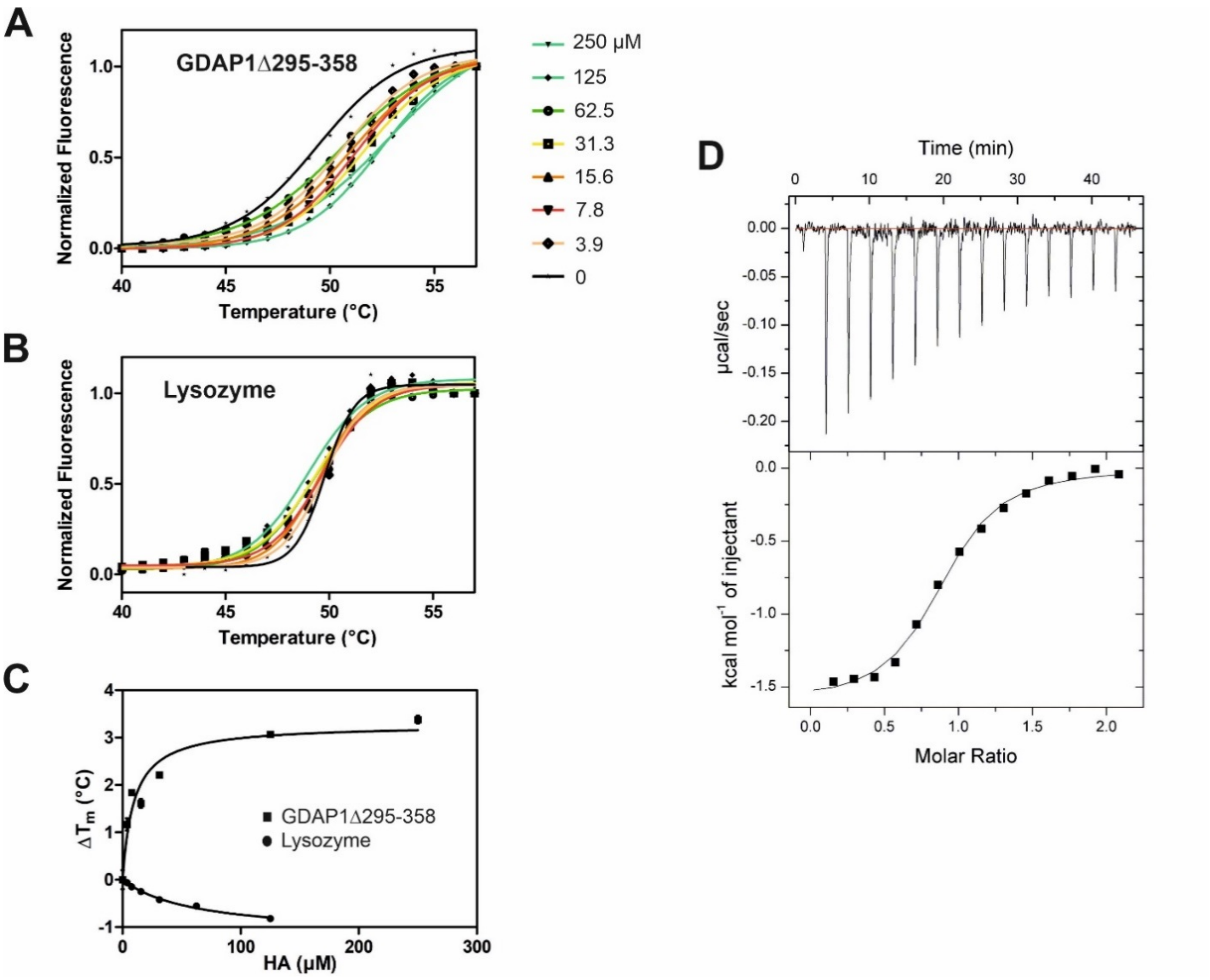

Supplementary Figure S1. HA binding stabilizes GDAP1Δ295-358. (A) Thermal unfolding data of GDAP1Δ295-358. (B) Thermal unfolding data of lysozyme as a control. (C) T_m shifts of GDAP1Δ295-358 upon HA titration. (D) ITC binding curve of HA binding to GDAP1Δ295-358.

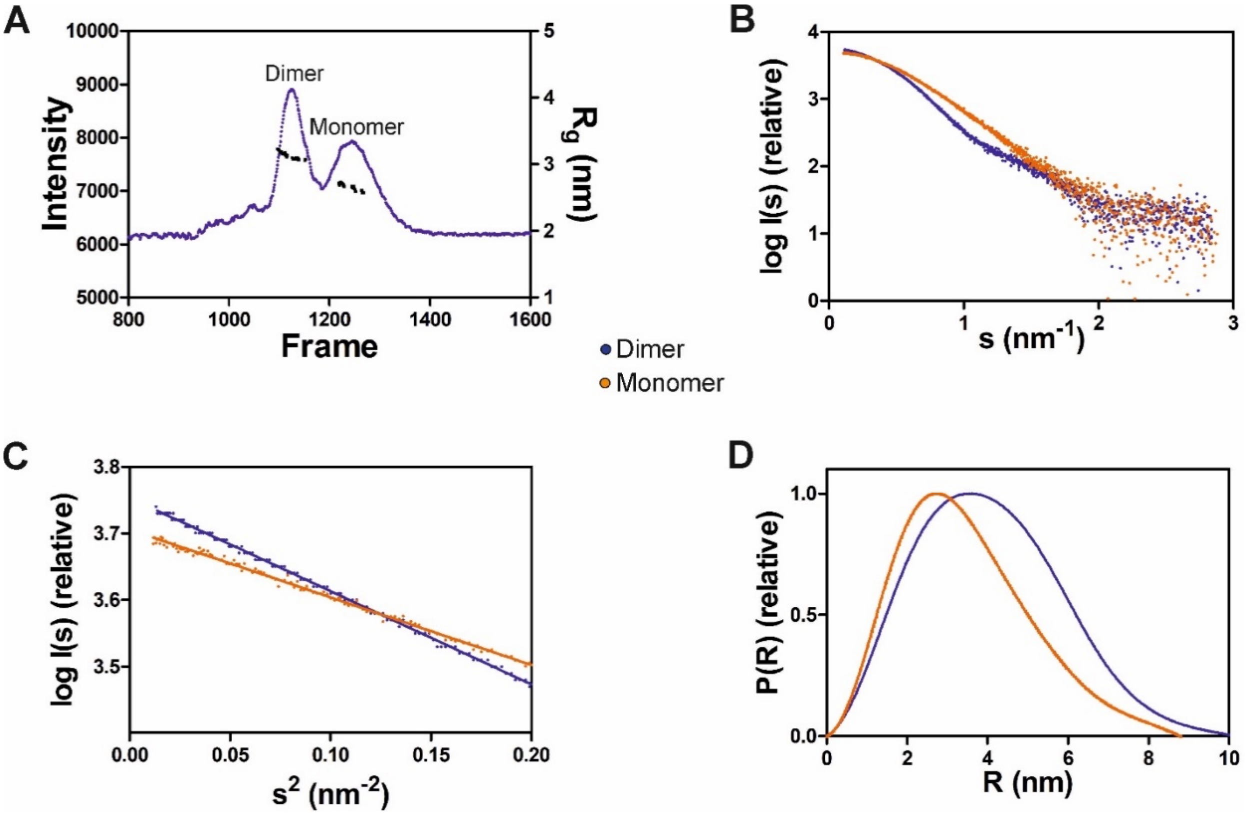

Supplementary Figure S2. SAXS analysis of GDAP1Δ295-358. (A) SEC-SAXS elution profile. R_g for the dimer and monomer peaks is also plotted. (B) Experimental scattering data (log (I_s) *vs*. s). (C) Guinier analysis. (D) Distance distribution function for dimer (blue) and monomer (orange).

-

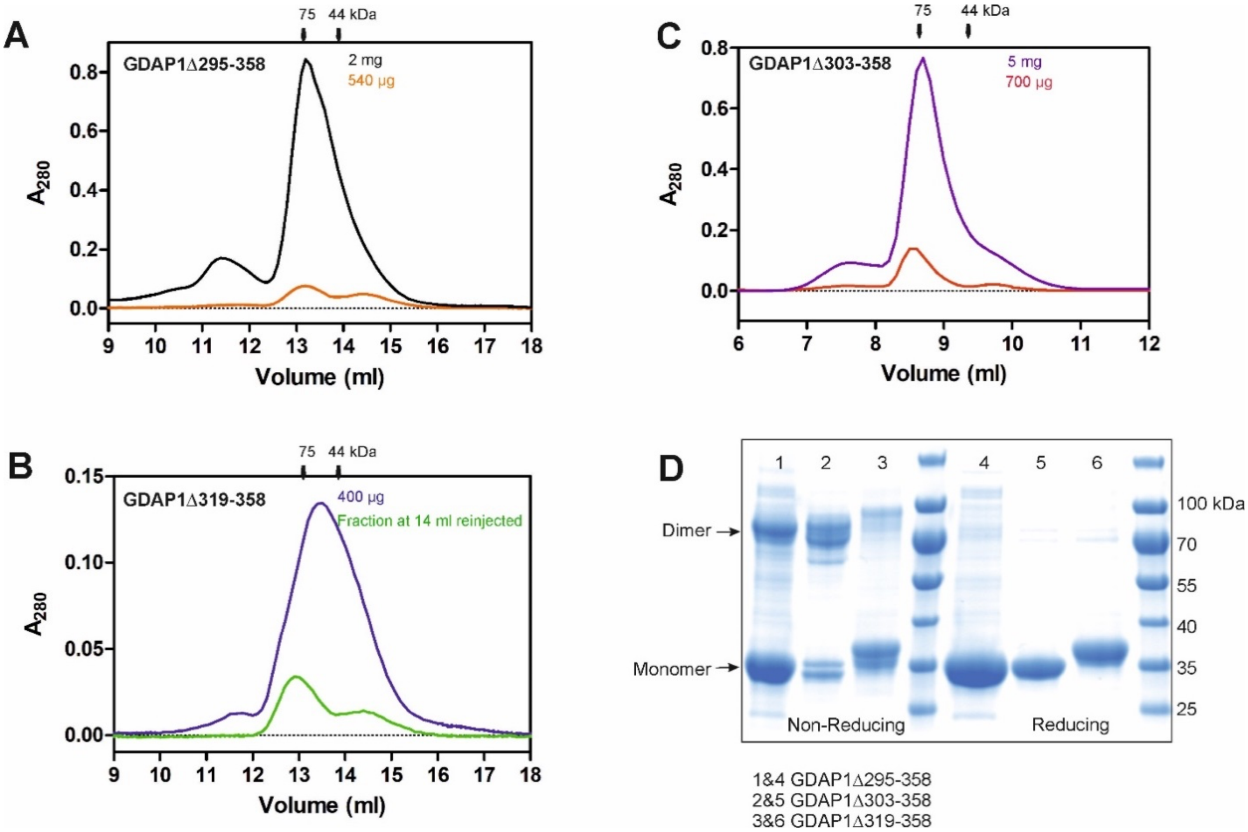

Supplementary Figure S3. SEC elution profiles and SDS-PAGE gels. The positions of the molecular weight markers are shown on top. (A) Elution profile of GDAP1Δ295-358 at 2 mg (black) and 540 µg (orange), column S200 increase 10/300 GL. (B) Elution profile of GDAP1Δ319-358 at 400 µg (blue) and ~100 µg (green), column S200 increase 10/300 GL. (C) Elution profile of GDAP1Δ303-358 at 5 mg (purple) and 700 µg (dark orange), column S75 increase 10/300 GL. (D) Non-reducing and reducing SDS-PAGE gel of three constructs.

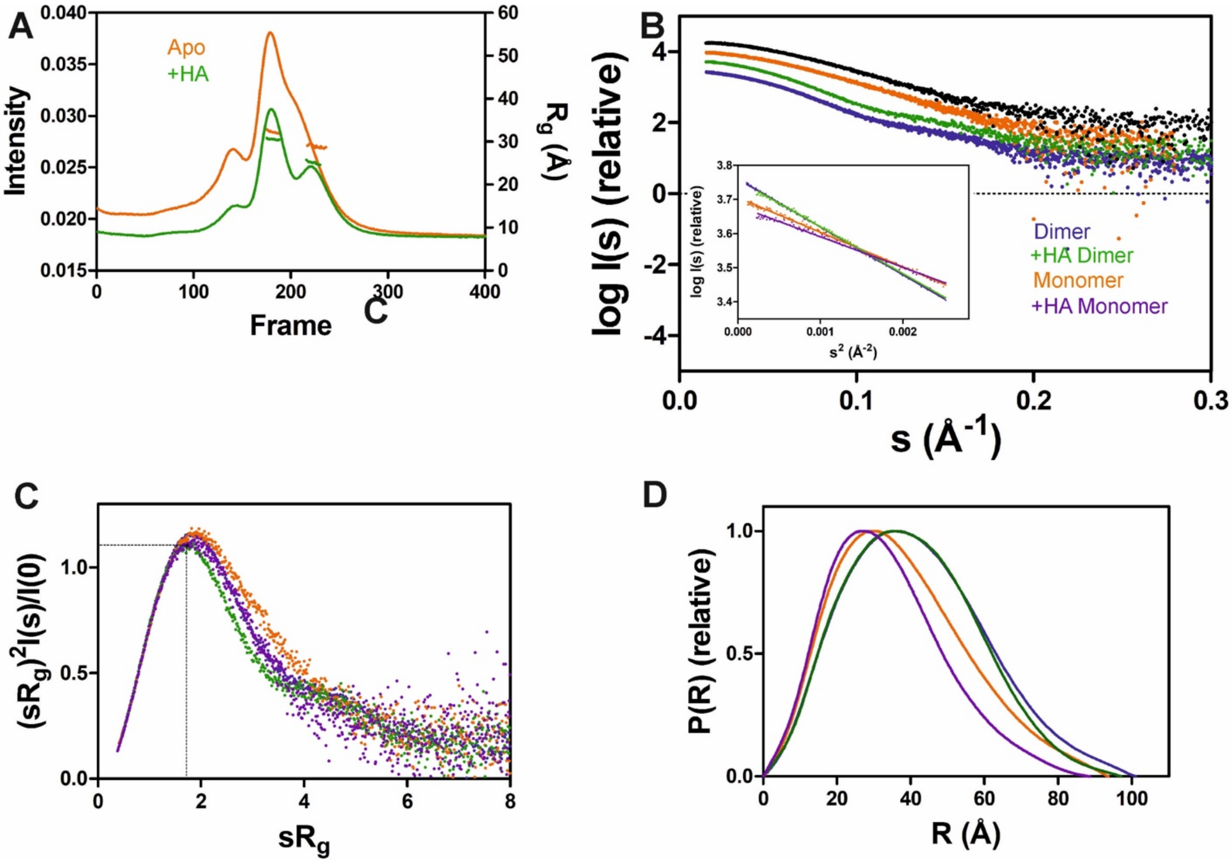

Supplementary Figure S4. SAXS analysis of GDAP1Δ295-358 in the absence and presence of HA. (A) In-line SEC-SAXS elution profiles and R_g plot of SAXS frames for dimer and monomer peaks of the protein. (B) Experimental scattering data (log (I_s) vs s) and Guinier analysis (inset). (C) R_g normalized Kratky plots, the dashed lines representing the maximum value of the standard globular protein. (D) Distance distributions *p(r)* plots of ligand-free GDAP1 dimer (blue) and monomer (orange), ligand-bound GDAP1 dimer (green) and monomer (purple).

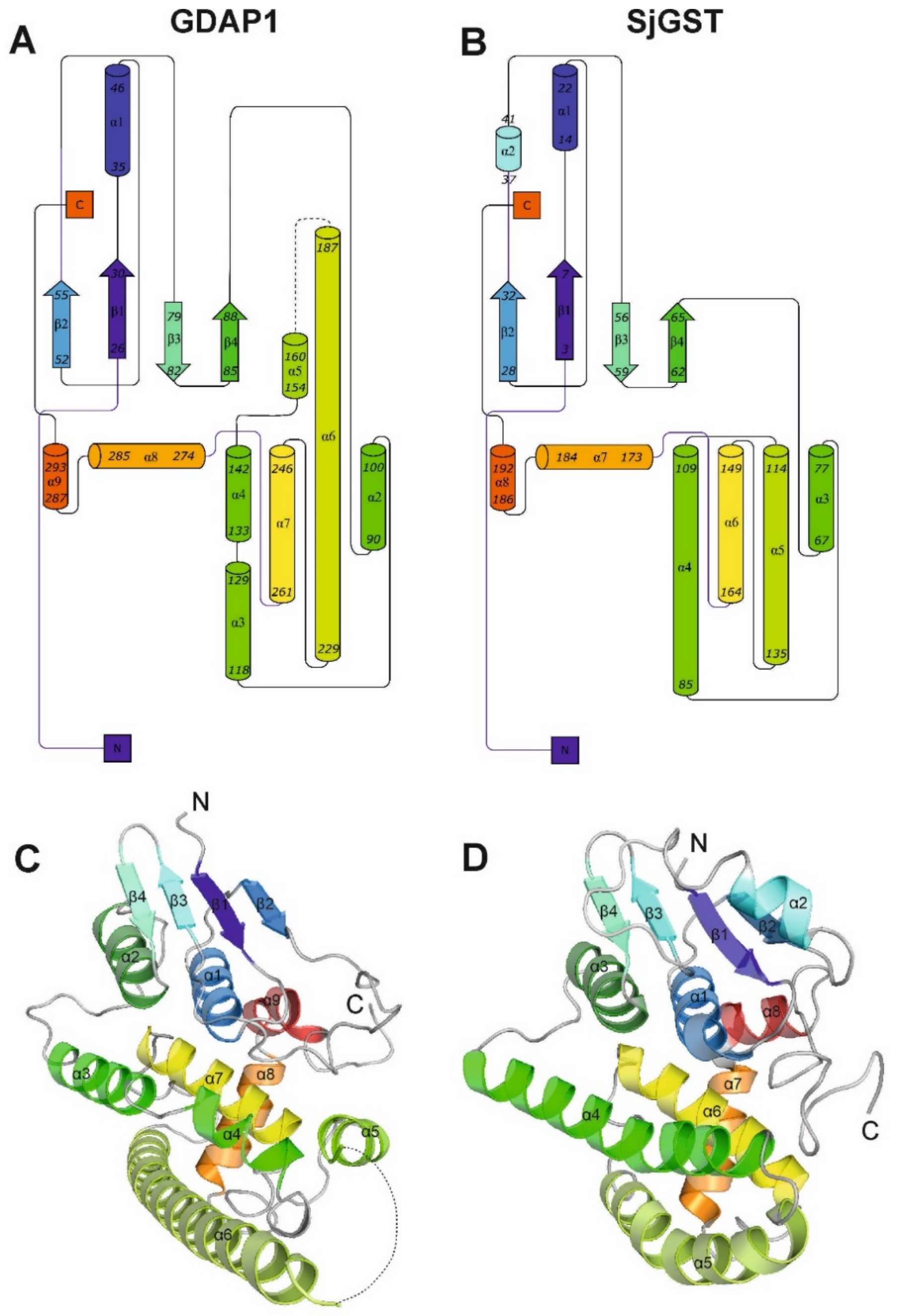

Supplementary Figure S5. Topology and structure of GDAP1 core domain and SjGST. (A,B) Topology diagrams for GDAP1Δ303-358 chain A and *Sj*GST (PDB:1UA5 (Kursula et al., 2005)). The dashed lines indicate loops not resolved in the electron density. **(**C,D) Cartoon representation of GDAP1 chain A and SjGST colored with gradient according to the topology diagrams. The dashed lines indicate loops not defined by electron density.

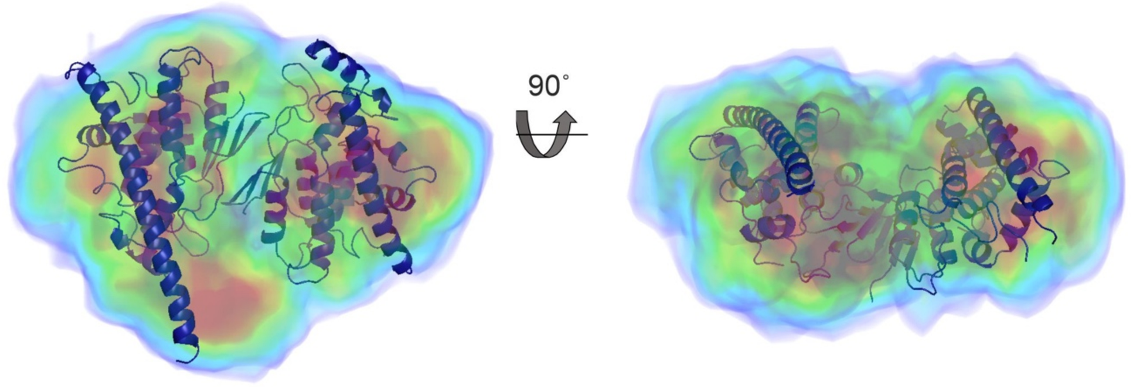

Supplementary Figure S6. Single particle electron density map reconstruction. Single particle electron density map reconstruction (multicolor volume) and crystal structure alignment (blue) calculation using DENSS. The mean of 20 iterative map calculations were performed including enantiomer search. The median fit to the scattering data χ^2= 1.455, and R_g= 32.1 Å. The calculated mean support volume of the particle was 197080.59 Å^3. The map resolution estimate was calculated with Fourier shell correlation function with a cut off value of 0.5. The mean resolution of 20 maps after refinement was 34.2 Å

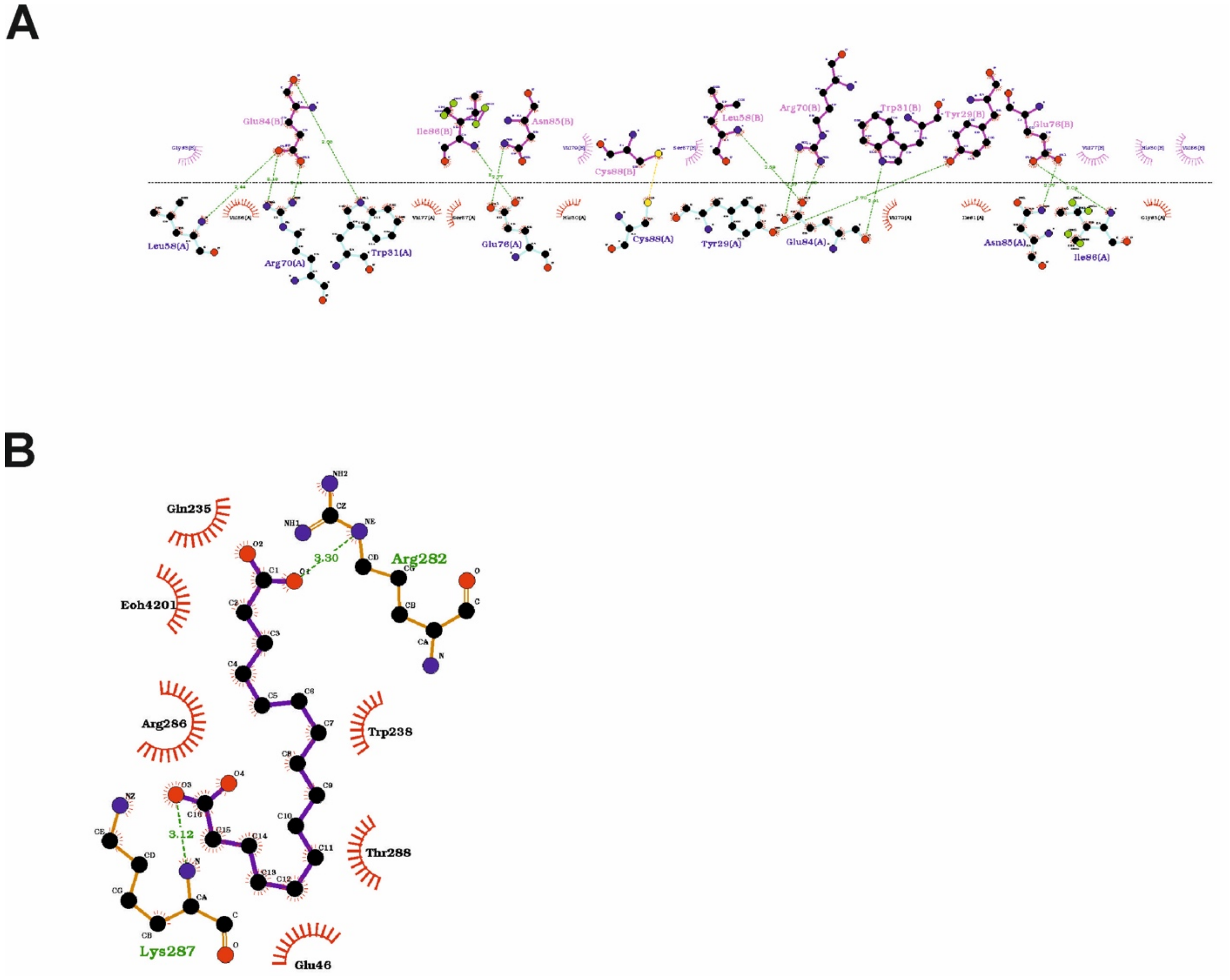

Supplementary Figure S7. Schematic view of interactions. (A) On the dimer interface. **(B)** between HA and GDAP1.

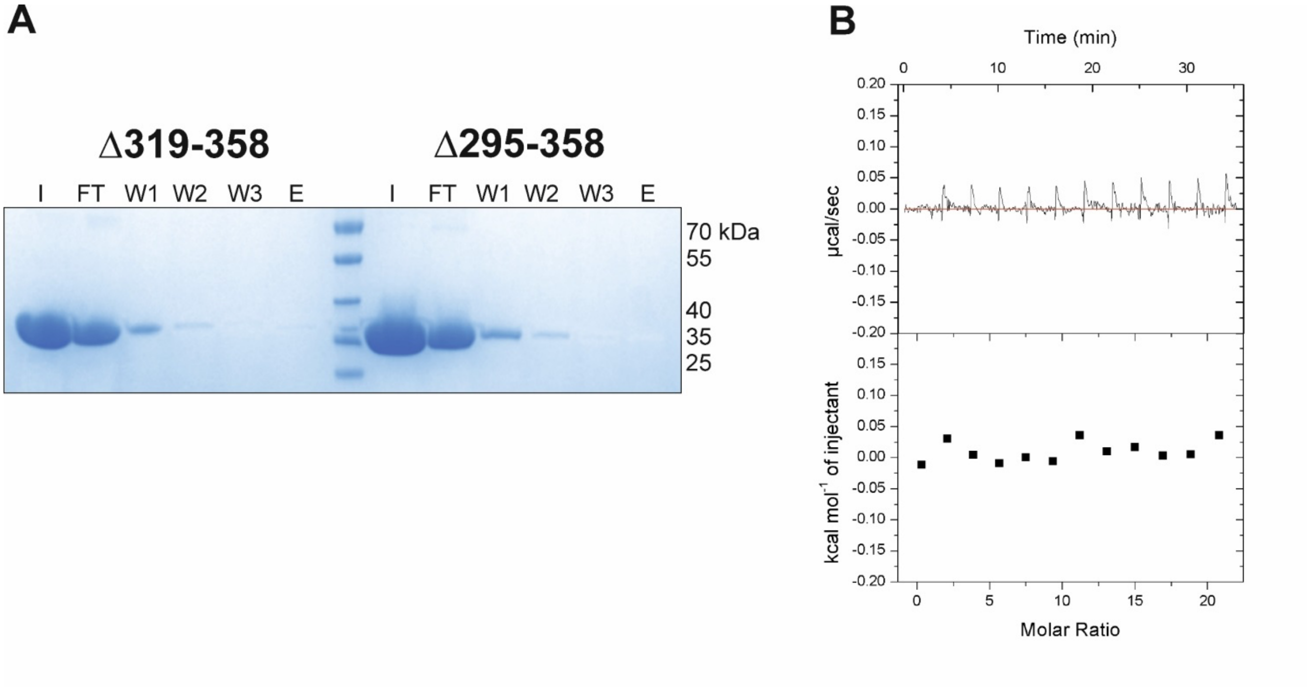

Supplementary Figure S8. GSH does not bind to GDAP1. (A) Reducing SDS-PAGE gel of GDAP1Δ319-358 and GDAP1Δ295-358 shows fractions collected in glutathione-sepharose binding assay. I: Input; FT: Flow through; W1, W2, W3: the first, second and third washing step, respectively; E: Elution**.** (B) 8 mM GSH was titrated into the cell containing 50 µM GDAP1Δ295-358 in an ITC experiment.

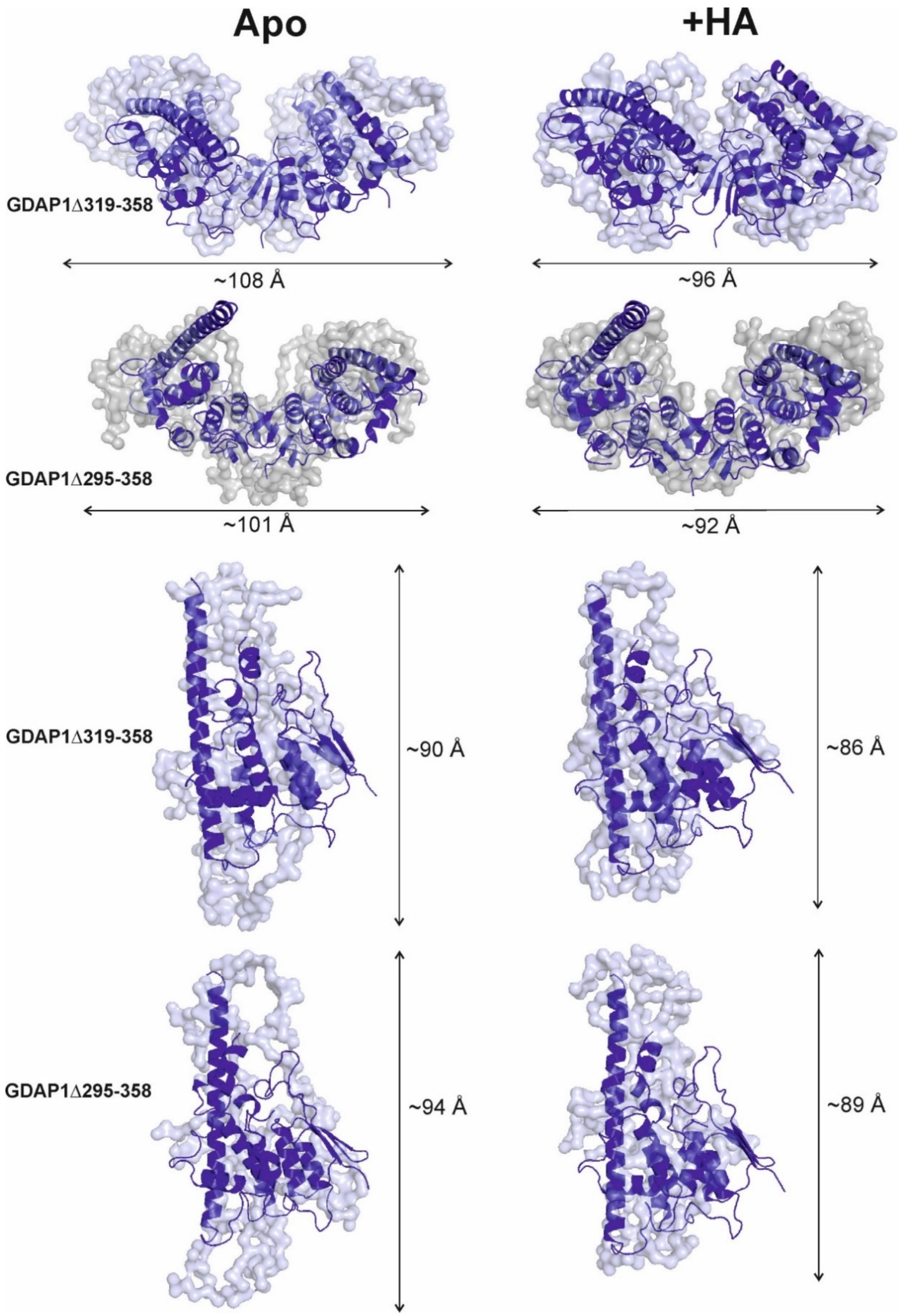

Supplementary Figure 9. Chain-like models of GDAP1Δ319-358 and GDAP1Δ295-358 dimer and monomer in the absence and presence of HA with the respective maximum dimensions

A B

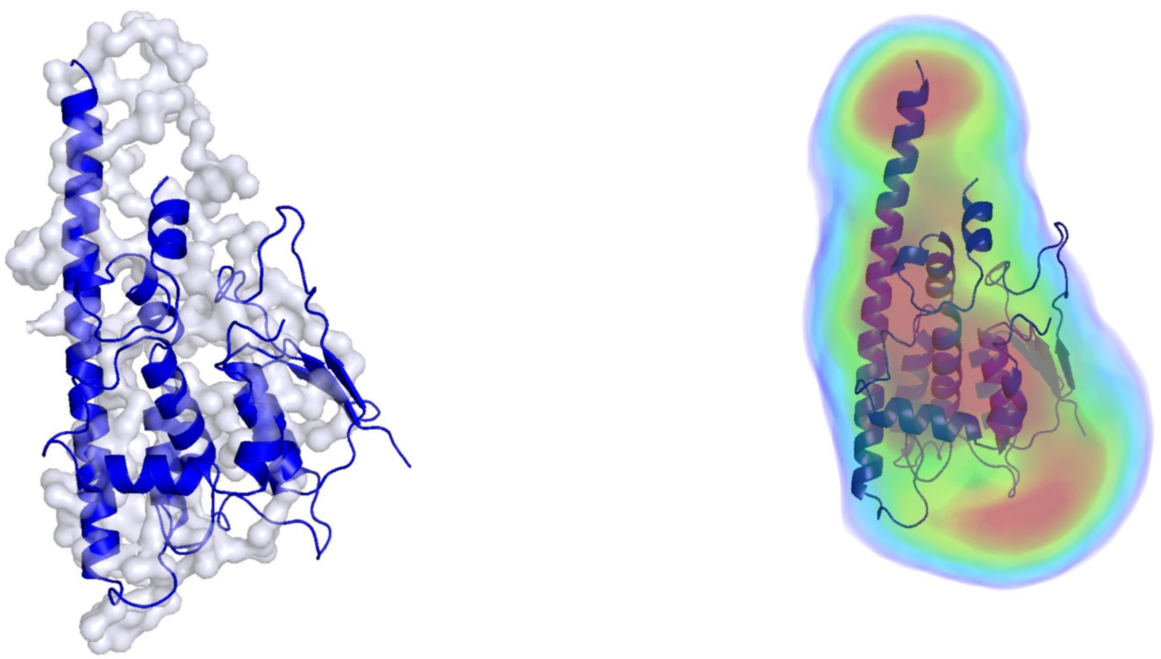

Supplementary Figure S10. *Ab initio* GASBOR models of mutant Y29E/C88A, and electron density map reconstruction calculated using DENSS. The Y29E/C88A GASBOR fit was χ^2= 1.301, electron density map median fit χ^2= 0.129, R_g= 26.3 and the map resolution estimate 45.6 Å where FSC=0.5.

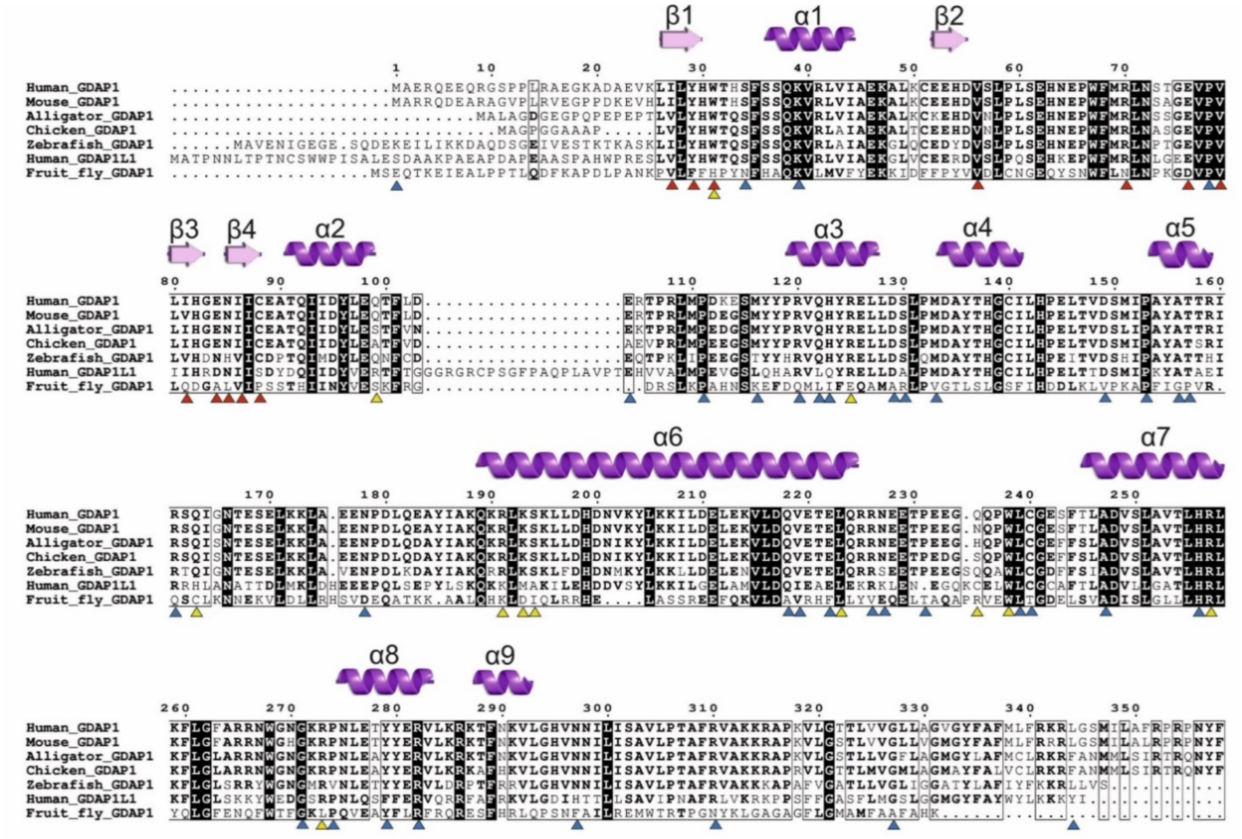

Supplementary Figure S11. Sequence alignment of GDAP1 and GDAP1L1. Important residues for dimer interface is highlighted in red triangles and those implicated in CMT missense and nonsense mutations (based on the human gene mutation database http://www.hgmd.cf.ac.uk/ac) are highlighted using the blue and yellow triangles, respectively.

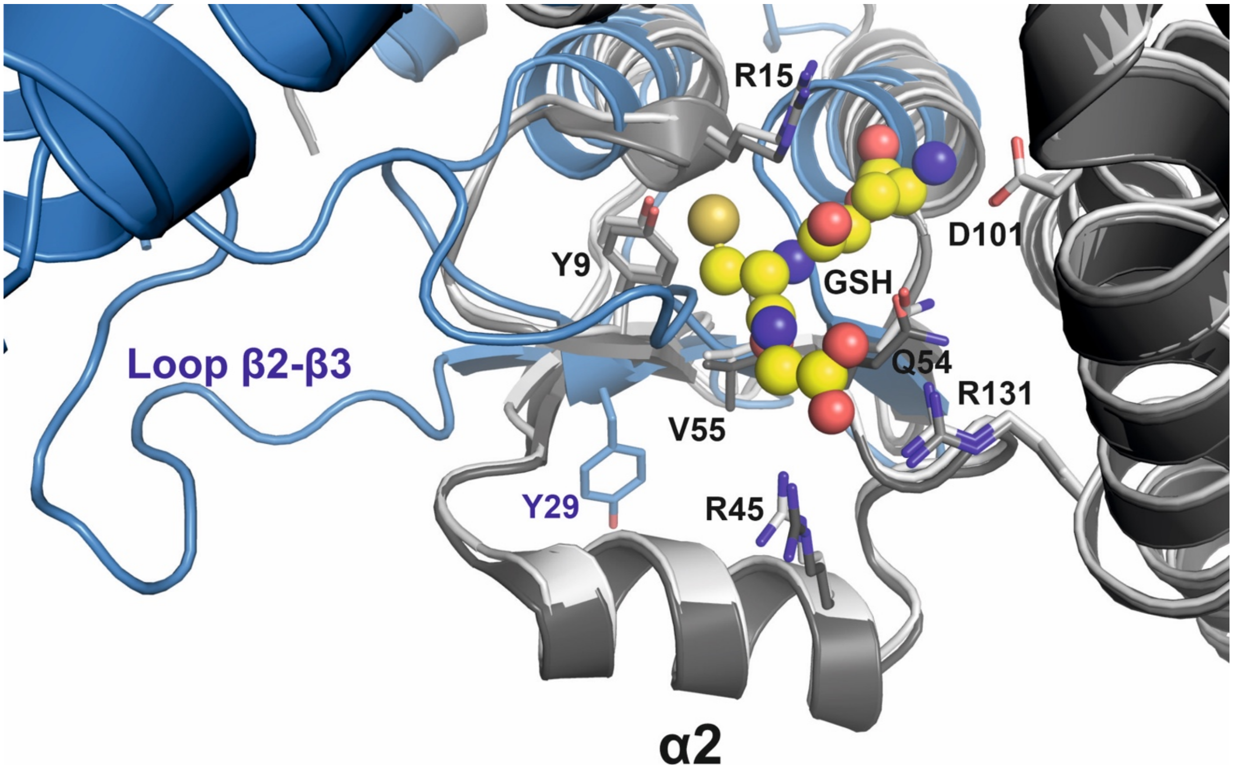

Supplementary Figure S12. Superposition of GDAP1 with GSTA1-1. Close-up view of the GDAP1 loop β2-β3 (blue) and GSTA1-1 α2 of apo (dark grey, PDB ID 1pkz) and GSH-bound (light-grey, PDB ID 1pkw) (Grahn et al., 2006). Key GST residues were shown in sticks and GSH was shown in spheres.

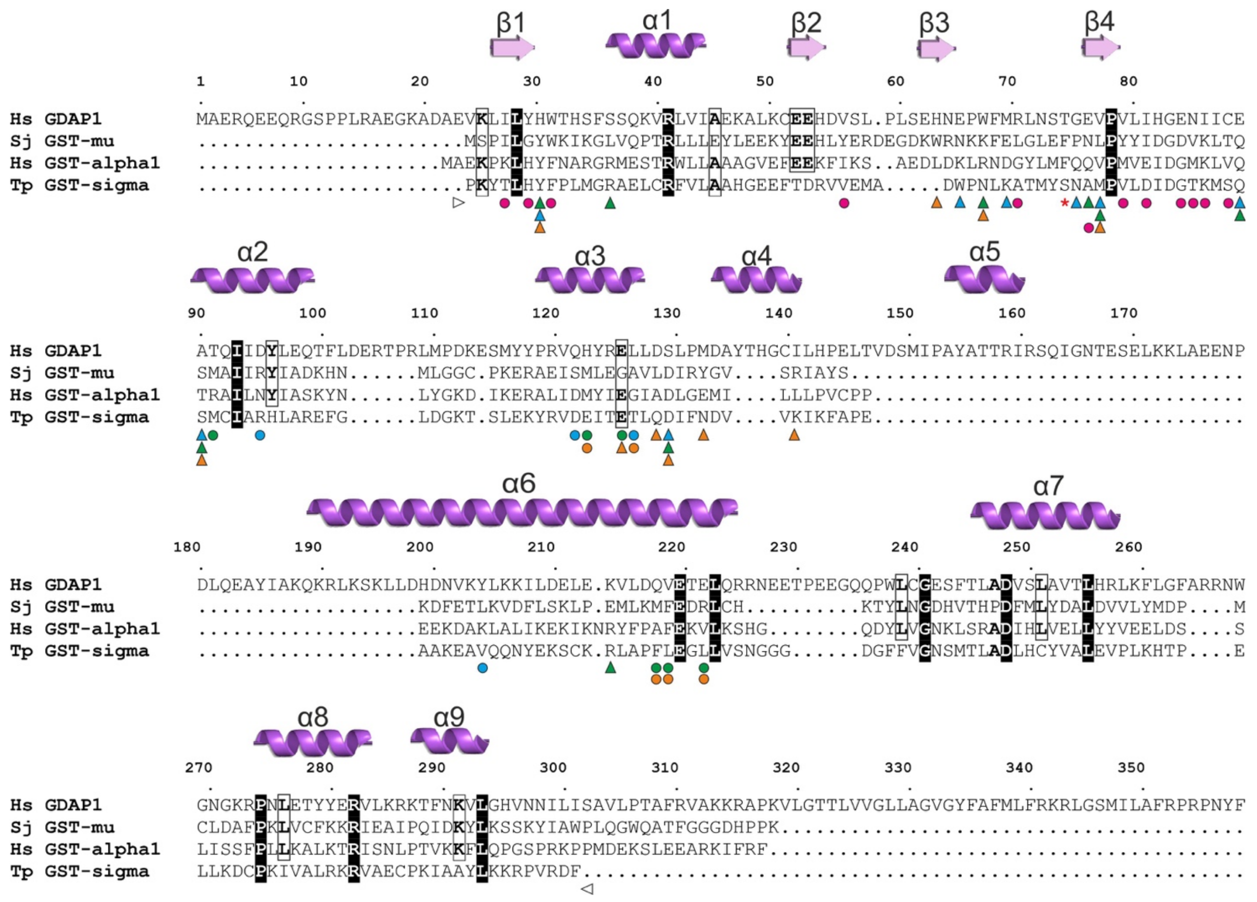

Supplementary Figure S13. Sequence alignment of generic GSTs and GDAP1 (PDB ID: 7ALM, secondary structure elements). The alignment highlights key substrate interacting residues in GSTs (triangles) and dimer interface residues in GDAP1 and GSTs (circles). Additionally, the "lock-and-key"-type binding pocket residue present in GSTs is annotated with a red asterisk. The range of the GDAP1 crystal structure is marked with sided white triangles. Annotations of the GSTs are marked as *Schistosoma japonicum* GST-mu with orange (PDB ID: 1UA5), *Homo sapiens* GST-A1 with green (PDB ID: 1PKW), and *Todarodes pacificus* GST sigma with cyan (PDB ID: 1GSQ), respectively. The GDAP1 dimer interface residues are annotated as purple circles.

2 Supplementary Tables

Supplementary Table S1. SASBDB entry codes

SASDJR8	Dimeric human ganglioside-induced differentiation-associated protein 1, construct GDAP1Δ295-358
SASDJS8	Dimeric human ganglioside-induced differentiation-associated protein 1, construct GDAP1Δ295-358 with hexadecanedioic acid
SASDJT8	Monomeric human ganglioside-induced differentiation-associated protein 1, construct GDAP1Δ295-358
SASDJU8	Monomeric human ganglioside-induced differentiation-associated protein 1, construct GDAP1Δ295-358 with hexadecanedioic acid
SASDJV8	Dimeric human ganglioside-induced differentiation-associated protein 1, construct GDAP1Δ303-358
SASDJW8	Monomeric human ganglioside-induced differentiation-associated protein 1, construct GDAP1Δ303-358, mutant Y29E/C88A
SASDJX8	Dimeric human ganglioside-induced differentiation-associated protein 1, construct GDAP1Δ319-358
SASDJY8	Dimeric human ganglioside-induced differentiation-associated protein 1, construct GDAP1Δ319-358 with hexadecanedioic acid
SASDJZ8	Monomeric human ganglioside-induced differentiation-associated protein 1, construct GDAP1Δ319-358
SASDJ29	Monomeric human ganglioside-induced differentiation-associated protein 1, construct GDAP1Δ319-358 with hexadecanedioic acid
SASDJ39	Monomeric human ganglioside-induced differentiation-associated protein 1-like 1, GDAP1L1

Supplementary Table S2. Electron density map reconstruction from SAXS scattering curves of wild-type and Y29E/C88A mutant GDAP1Δ303-358

Structural parameters	WT	Y29E/C88A
**s-range (s = 4π sin(θ)/λ)**	0-0.504	0-0.327
**2-fold symmetry**	Yes	No
**D_{max} allowed (Å)**	96	91
**Real space box width/range (Å)**	228/±144	234/±117.5
**Real space box volume (Å^3)**	23887872	12966281
**Real space voxel size (Å)**	4.5	3.6
**Real space voxel volume (Å^3)**	91.1	49.5
**Chi^2**	1.455	1.301
**R_g (Å)**	32.1	26.3
**Map resolution (FCS= 0.5)**	34.2	45.6

Supplementary Table S3. GST activity measurement

	CDNB (340 nm)				NBC (360 nm)				EPNP (310 nm)	
	**k2 (s^{-1})**	**±**	**Specific activity (nmol/min/µg)**	**±**	**k2 (s^{-1})**	**±**	**Specific activity (nmol/min/µg)**	**±**	**k2 (s^{-1})**	**Specific activity (nmol/min/µg)**
**GST**	11.7	1.55	26.1	8.5	0.12	0.06	0.27	0.075	n.d	n.d
**GDAP1∆295-358**	7E-04	8E-04	2E-02	2E-02	6.005E-04	1.147E-04	1.334E-03	2.549E-04	n.d	n.d
**GDAP1∆303-358**	8.89E-03	1.25E-03	1.98E-02	2.77E-03	2.553E-03	1.686E-03	5.673E-03	3.747E-03	n.d	n.d
**GDAP1∆319-358**	3.81E-03	8.52E-05	6.35E-04	1.42E-05	1.631E-03	1.416E-03	3.625E-03	3.146E-03	n.d	n.d

3 References

Grahn, E., Novotny, M., Jakobsson, E., Gustafsson, A., Grehn, L., Olin, B., Madsen, D., Wahlberg, M., Mannervik, B., Kleywegt, G.J. (2006). New crystal structures of human glutathione transferase A1-1 shed light on glutathione binding and the conformation of the C-terminal helix. *Acta Crystallogr D Biol Crystallogr* 62, 197–207. DOI:10.1107/S0907444905039296

Kursula, I., Heape, A.M., Kursula, P. (2005). Crystal structure of non-fused glutathione S-transferase from Schistosoma japonicum in complex with glutathione. *Protein Pept Lett* 12, 709–712. DOI:10.2174/0929866054696154
